# Structural Properties of the *Caenorhabditis elegans* Neuronal Network

**DOI:** 10.1371/journal.pcbi.1001066

**Published:** 2011-02-03

**Authors:** Lav R. Varshney, Beth L. Chen, Eric Paniagua, David H. Hall, Dmitri B. Chklovskii

**Affiliations:** 1 Massachusetts Institute of Technology, Cambridge, Massachusetts, United States of America; 2 Cold Spring Harbor Laboratory, Cold Spring Harbor, New York, United States of America; 3 California Institute of Technology, Pasadena, California, United States of America; 4 Albert Einstein College of Medicine, Bronx, New York, United States of America; 5 Howard Hughes Medical Institute, Janelia Farm Research Campus, Ashburn, Virginia, United States of America; Indiana University, United States of America

## Abstract

Despite recent interest in reconstructing neuronal networks, complete wiring diagrams on the level of individual synapses remain scarce and the insights into function they can provide remain unclear. Even for *Caenorhabditis elegans*, whose neuronal network is relatively small and stereotypical from animal to animal, published wiring diagrams are neither accurate nor complete and self-consistent. Using materials from White *et al.* and new electron micrographs we assemble whole, self-consistent gap junction and chemical synapse networks of hermaphrodite *C. elegans*. We propose a method to visualize the wiring diagram, which reflects network signal flow. We calculate statistical and topological properties of the network, such as degree distributions, synaptic multiplicities, and small-world properties, that help in understanding network signal propagation. We identify neurons that may play central roles in information processing, and network motifs that could serve as functional modules of the network. We explore propagation of neuronal activity in response to sensory or artificial stimulation using linear systems theory and find several activity patterns that could serve as substrates of previously described behaviors. Finally, we analyze the interaction between the gap junction and the chemical synapse networks. Since several statistical properties of the *C. elegans* network, such as multiplicity and motif distributions are similar to those found in mammalian neocortex, they likely point to general principles of neuronal networks. The wiring diagram reported here can help in understanding the mechanistic basis of behavior by generating predictions about future experiments involving genetic perturbations, laser ablations, or monitoring propagation of neuronal activity in response to stimulation.

## Author Summary

Connectomics, the generation and analysis of neuronal connectivity data, stands to revolutionize neurobiology just as genomics has revolutionized molecular biology. Indeed, since neuronal networks are the physical substrates upon which neural functions are carried out, their structural properties are intertwined with the organization and logic of function. In this paper, we report a near-complete wiring diagram of the nematode *Caenorhabditis elegans* and present several analyses of its properties, finding many nonrandom features. We give novel visualizations and compute network statistics to enhance understanding of the reported data. We also use principled systems-theoretic methods to generate hypotheses on how biological function may arise from the reported neuronal network structure. The wiring diagram reported here can further be used to generate predictions about signal propagation in future perturbation, ablation, or artificial stimulation experiments.

## Introduction

Determining and examining base sequences in genomes [1], [2] has revolutionized molecular biology. Similarly, decoding and analyzing connectivity patterns among neurons in nervous systems, the aim of the emerging field of connectomics [3]–[6], may make a major impact on neurobiology. Knowledge of connectivity wiring diagrams alone may not be sufficient to understand the function of nervous systems, but it is likely necessary. Yet because of the scarcity of reconstructed connectomes, their significance remains uncertain.

The neuronal network of the nematode *Caenorhabditis elegans* is a logical model system for advancing the connectomics program. It is sufficiently small that it can be reconstructed and analyzed as a whole. The 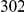 neurons in the hermaphrodite worm are identifiable and consistent across individuals [7]. Moreover the connections between neurons, consisting of chemical synapses and gap junctions, are stereotypical from animal to animal with more than 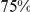 reproducibility [7]–[10].

Despite a century of investigation [11], [12], knowledge of nematode neuronal networks is incomplete. The basic structure of the *C. elegans* nervous system had been reconstructed using electron micrographs [7], but a major gap in the connectivity of ventral cord neurons remained. Previous attempts to assemble the whole wiring diagram made unjustified assumptions that several reconstructed neurons were representative of others [13]. Much previous work analyzed the properties of the neuronal network (see e.g. [14]–[20] and references therein and thereto) based on these incomplete or inconsistent wiring diagrams [7], [13].

In this paper, we advance the experimental phase of the connectomics program [6], [21] by reporting a near-complete wiring diagram of *C. elegans* based on original data from White *et al.* [7] but also including new serial section electron microscopy reconstructions and updates. Although this new wiring diagram has not been published definitively before now, it has already been freely shared with the community through the WormAtlas [22] and has also been used in previous studies such as [23]. See Methods section for further details on the wiring diagram and on freely obtaining it in electronic form.

We advance the theoretical phase of connectomics [24], [25], by characterizing signal propagation through the reported neuronal network and its relation to behavior. We compute for the first time, local properties that may play a computational purpose, such as the distribution of multiplicity and the number of terminals, as well as global network properties associated with the speed of signal propagation. Unlike the conventional “hypothesis-driven” mode of biological research, our work is primarily “hypothesis-generating” in the tradition of systems biology.

Our results should help investigate the function of the *C. elegans* neuronal network in several ways. A full wiring diagram, especially when conveniently visualized using a method proposed here, helps in designing maximally informative optical ablation [26] or genetic inactivation [27] experiments. Our eigenspectrum analysis characterizes the dynamics of neuronal activity in the network, which should help predict and interpret the results of experiments using sensory and artificial stimulation and imaging of neuronal activity.

Organization of the Results section reflects the duality of contribution and follows the tradition laid down by genome sequencing [1], [2]. We start by describing and visualizing the wiring diagram. Next, we analyze the non-directional gap junction network and the directional chemical synapse network separately. We perform these analyses separately because understanding the parts before the whole provides didactic benefits and because this delays making assumptions about the relative weight of gap junctions and chemical synapses. Finally, we analyze the combined network of gap junctions and chemical synapses.

## Results

### Reconstruction

#### An updated wiring diagram

The *C. elegans* nervous system contains 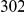 neurons and is divided into the pharyngeal nervous system containing 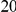 neurons and the somatic nervous system containing 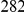 neurons. We updated the wiring diagram (see Methods) of the larger somatic nervous system. Since neurons CANL/R and VC06 do not make synapses with other neurons, we restrict our attention to the remaining 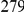 somatic neurons. The wiring diagram consists of 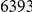 chemical synapses, 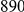 gap junctions, and 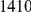 neuromuscular junctions.

The new version of the wiring diagram incorporates original data from White *et al.* [7], Hall and Russell [10], updates based upon later work [8], [Hobert O and Hall DH, unpublished], as well as new reconstructions; see Methods for details. In total, over 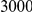 synaptic contacts, including chemical synapses, gap junctions, and neuromuscular junctions were either added or updated from the previous version of the *C. elegans* wiring diagram.

The current wiring diagram is considered self-consistent under the following criteria:

1. A record of Neuron 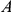 sending a chemical synapse to Neuron 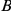 must be paired with a record of Neuron 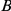 receiving a chemical synapse from Neuron 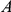.
2. A record of gap junction between Neuron 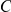 and Neuron 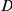 must be paired with a separate record of gap junction between Neuron 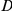 and Neuron 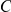.

Although the updated wiring diagram represents a significant advance, it is only about 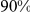 complete because of missing data and technical difficulties. Due to sparse sampling along lengths of the sublateral, canal-associated lateral, and midbody dorsal cords, about 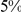 of the total chemical synapses are missing, as concluded from antibody staining for synapses [Duerr JS, Hall DH, and Rand JB, unpublished]. Many gap junctions are likely missing due to the difficulty in identifying them in electron micrographs using conventional fixation and imaging methods. Hopefully, application of high-pressure freezing techniques and electron tomography will help identify missing gap junctions [28]. Finally, it should be noted that this reconstruction combined partial imaging of three worms, with images for the posterior midbody being from the male *N2Y*.

The basic qualitative properties of the updated *C. elegans* nervous system remain as reported previously [7]–[9]. Neurons are divided into 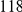 classes, based on morphology, dendritic specialization, and connectivity. Based on neuronal structural and functional properties, the classes can be divided into three categories: sensory neurons, interneurons, and motor neurons. Neurons known to respond to specific environmental conditions, either anatomically, by sensory ending location, or functionally, are classified as sensory neurons. They constitute about a third of neuron classes. Motor neurons are recognized by the presence of neuromuscular junctions. Interneurons are the remainder of the neuron classes and constitute about half of all classes. A few of the neurons could have dual classification, such as sensory/motor neurons. Some interneurons are much more important for developmental function than for function in the final neuronal network [28].

The majority of sensory neuron and interneuron categories contain pairs of bilaterally symmetric neurons. Motor neurons along the body are organized in repeating groups whereas motor neurons in the head have four- or six-fold symmetry. A large fraction of neurons send long processes to the nerve ring in the circumpharyngeal region to make synapses with other neurons [7].

The neurons in *C. elegans* are structurally simple: most neurons have one or two unbranched processes and form *en passant* synapses along them. Dendrites are recognized by being strictly “postsynaptic” or by containing a specialized sensory apparatus, such as amphid and phasmid sensory neurons. Interneurons lack clear dendritic specialization. It is interesting to note that a given worm neuron has connections with only about 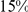 of neurons with which it has physical contact [7], [8], a similar number to the connectivity fraction in other nervous systems [29], [30].

#### Wiring diagram as adjacency matrices

**Figure 1:**
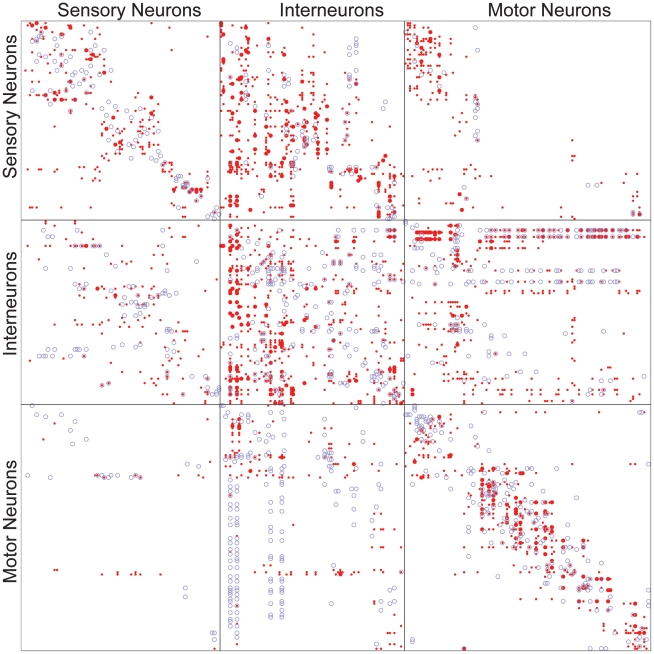
Adjacency matrices for the gap junction network (blue circles) and the chemical synapse network (red points) with neurons grouped by category (sensory neurons, interneurons, motor neurons). Within each category, neurons are in anteroposterior order. Among chemical synapse connections, small points indicate less than 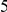 synaptic contacts, whereas large points indicate 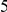 or more synaptic contacts. All gap junction connections are depicted in the same way, irrespective of number of gap junction contacts.

In the remainder of the paper, we describe and analyze the connectivity of gap junction and chemical synapse networks of *C. elegans* neurons. Gap junctions are channels that provide electrical coupling between neurons, whereas chemical synapses use neurotransmitters to link neurons. The network of gap junctions and the network of chemical synapses are initially treated separately, with each represented by its own adjacency matrix, Figure 1. In an adjacency matrix 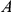, the element in the 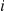
th row and 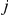
th column, 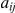, represents the total number of synaptic contacts from the 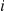
th neuron to the 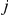
th. If neurons are unconnected, the corresponding element of the adjacency matrix is zero. An adjacency matrix may be used due to self-consistency in the gathered data.

Although gap junctions may have directionality, i.e. conduct current in only one direction, this has not been demonstrated in *C. elegans*. Even if directionality existed, such information cannot be extracted from electron micrographs. Thus we treat the gap junction network as an undirected network with a symmetric adjacency matrix, as depicted in Figure 1. Weights in both 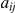 and 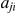 represent the total number of gap junctions between neurons 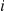 and 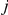.

Since chemical synapses possess clear directionality that can be extracted from electron micrographs, we represent the chemical network as a directed network with an asymmetric adjacency matrix, Figure 1. The elements of the adjacency matrix take nonnegative values, which reflect the number of synaptic contacts between corresponding neurons. Contacts are given equal weight, regardless of the apparent size of the synaptic apposition. We use nonnegative values for most of the paper because we cannot determine whether a synapse is excitatory, inhibitory, or modulatory from electron micrographs of *C. elegans*. For the linear systems analysis, we do however make a rough guess of the signs of synapses based on neurotransmitter gene expression data.

Electron micrographs for *C. elegans* have a further limitation that causes some synaptic ambiguity. When a presynaptic terminal makes contact with two adjacent processes of different neurons (send_joint in Durbin's notation [8]), it is not known which of these processes acts as a postsynaptic terminal; both might be involved. We count all polyadic synaptic connections. Polyadic connections are briefly revisited in the Discussion.

#### Visualization

**Figure 2:**
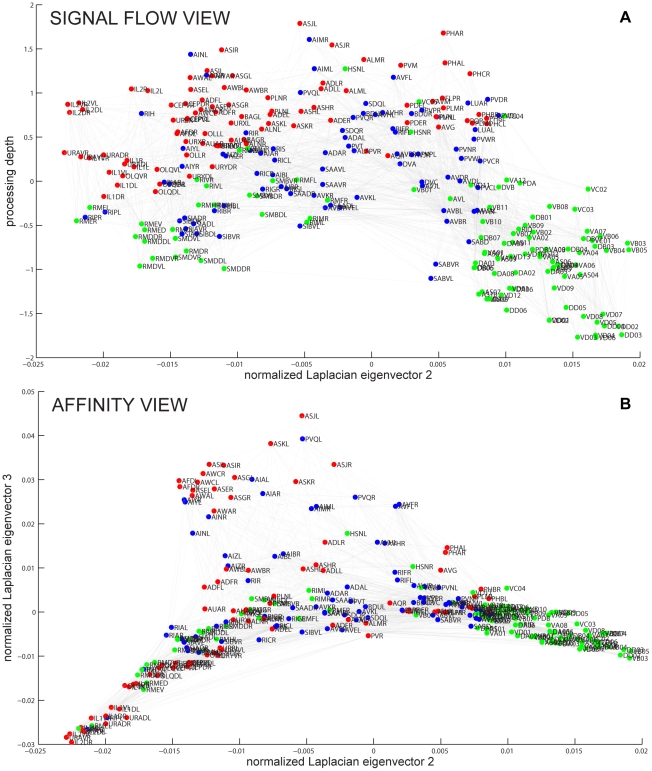
The *C. elegans* wiring diagram is a network of identifiable, labeled neurons connected by chemical and electrical synapses. Red, sensory neurons; blue, interneurons; green, motorneurons. (a). Signal flow view shows neurons arranged so that the direction of signal flow is mostly downward. (b). Affinity view shows structure in the horizontal plane reflecting weighted non-directional adjacency of neurons in the network.

Although statistical measures that we investigate later in this paper provide significant insights, they are no substitute to exploring detailed connectivity in the neuronal network. As the number of connections between neurons is large even for relatively simple networks, such analysis requires a convenient way to visualize the wiring diagram. Previously, various fragments of the wiring diagram were drawn to illustrate specific pathways [8], [31], [32]. Here, we propose a method to visualize the whole wiring diagram in a way that reflects signal flow through the network as well as the closeness of neurons in the network, Figure 2. To this end, we use spectral network drawing techniques because they have certain optimality properties [33] and aesthetic appeal. Next, we give an intuitive description of our visualization method; mathematical details can be found in Text S1.

The vertical axis in Figure 2(a), represents the position of neurons in the signal flow hierarchy [34], [35] of the chemical synapse network with sensory neurons at the top and motor neurons at the bottom, with interneurons in between. We want the vertical coordinate of pre- and post-synaptic neurons to differ by one, however due to “frustration” this is not always possible. Frustration happens when distances measured along network connections cannot be made to correspond to the hierarchy distances: there are two different hierarchical paths that require a particular neuron to appear in two different places. We look for the layout that has smallest deviation from this condition and find a closed form solution [34], [36].

The distance along the vertical coordinate corresponds roughly to the number of synapses from sensory to motor neurons—the signal flow depth of the network. Depending on the specific neurons considered, the distance from a sensory neuron to a motor neuron is 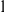
–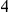. At the same time, the minimum number of chemical synapses crossed from sensory to motor neuron averaged over all such pairs is 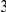, see also [8].

Neuronal position on the horizontal plane, Figure 2(b), represents the connectivity closeness of neurons in the combined chemical and electrical synapse network. Neuronal coordinates are given by the second and third eigenmodes of the symmetrized network's graph Laplacian (see below). In this representation, pairs of synaptically coupled neurons with larger number of connections in parallel tend to be positioned closer in space.

Thus, Figure 2 represents not the physical placement of neurons in the worm but signal flow and closeness in the network. Such visualization reveals that motorneurons and some interneurons segregate into two lobes along the first horizontal axis: the right lobe contains motorneurons in the ventral cord and the left lobe consists of neck neurons. The bi-lobe structure suggests partial autonomy of motorneurons in the ventral cord and neck. Interneurons that could coordinate the function of the two lobes can be easily identified by their central location.

### Gap Junction Network

For quantitative characterization, we first consider the gap junction network.

#### Basic structure and connectivity

The gap junction network that we analyze consists of 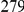 neurons and 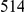 gap junction connections, consisting of one or more junctions. The network is not fully connected, but is divided into a giant component containing 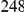 neurons, two smaller components of 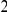 and 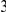 neurons, and 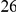 isolated neurons with no gap junctions (Table 1 in Text S4). The giant component has 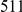 connections. Using connectivity data from [13], Majewska and Yuste had previously pointed out that most neurons in *C. elegans* belong to the giant component [37]. Our results agree roughly with [37], although our dataset excludes non-neuronal cells and places certain neurons in different connected components.

To evaluate the significance of the number of neurons in the giant component, we compare it with those expected in random networks. We start with the Erdös-Rényi random network because its construction requires a single parameter, the probability of a connection between two neurons. An Erdös-Rényi random network with 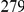 neurons and connection probability 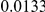 (thus with 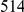 expected connections) would be expected to have 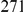 neurons in the giant component. The true gap junction giant component is much smaller; the probability of finding such a small giant component in a random network is on the order of 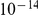 (see Methods).

A better comparison, however, can be made to random networks with degree distributions that match the degree distribution of the gap junction network [38]. Here, the degree of a neuron is the number of neurons with which it makes a gap junction. The giant component in a degree-matched random network would be expected to be 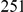 neurons (see Methods), about the same size as the measured giant component.

We may explore the utility of representing the wiring diagram as a three-layer network by grouping neurons by category (sensory neurons, interneurons, motor neurons). As shown in Tables 2A and 2B in Text S4, each category has many recurrent connections within and between categories (with the exception of connections between sensory and motor neurons). In particular, Table 2B in Text S4 indicates that motor neurons send back to interneurons roughly the same number of connections as they recurrently sent back to motor neurons. These observations suggest that when considering only gap junctions, a three-layer network abstraction may not be particularly useful.

#### Distributions of degree, multiplicity and the number of terminals

In this section, we analyze statistical properties of individual neurons and synaptic connections. To characterize the ability of individual neurons to propagate or collect signals, we compute the degree 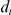 of neuron 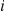, which is the number of neurons that are coupled to 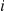 by at least one gap junction. The mean degree is 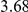, however this value is not representative as the degree varies in a wide range, from 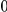 to 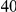. Thus, it is important to look at the degree distribution, which has been used to characterize and classify other networks previously [39]–[42].

**Figure 3:**
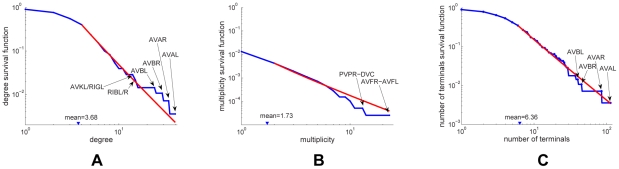
Survival functions for the distributions of degree, multiplicity, and number of synaptic terminals in the gap junction network. Neurons or connections with exceptionally high statistics are labeled. The tails of the distributions can be fit by a power law with the exponent 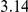 for the degrees (a); 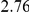 for the multiplicity distribution (b); 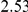 for the number of synaptic terminals (c). The exponents for the power law fits of the corresponding survival functions are obtained by subtracting one.

To visualize the discrete degree distribution, 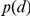, we use the survival function:
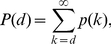
(1)which is the complement of the cumulative distribution function, Figure 3(a). The advantages of looking at the survival function rather than the degree distribution directly are that histogram binning is not required and that noise in the tail is reduced [43]. The survival function is also later applied to visualize other statistics. Various commonly encountered distributions and their corresponding survival functions are given in Text S2.

We perform a fitting procedure for the tail of the gap junction degree distribution [42] (see Methods). We find that the tail (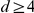) can be fit by the power law with exponent 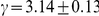), Figure 3(a), but not by the exponential decay (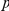-value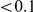). This result is consistent with the view that the gap junction network is scale-free [40].

To characterize the direct impact that one neuron can have on another, we quantify the strength of connections by the multiplicity, 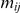, between neurons 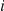 and 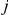, which is the number of synaptic contacts (here gap junctions) connecting 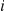 to 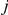. The degree treats synaptic connections as binary, whereas the multiplicity, also called edge weight, quantifies the number of contacts. The multiplicity distribution for the gap junction network is shown in Figure 3(b). We find that the multiplicity distribution for 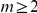 obeys a power law with exponent 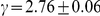). Although the exponential decay fit to the tail passes the 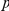-value test, the log-likelihood is significantly lower than for the power law.

Finally, the sum of the multiplicities of all gap junction connections of a given neuron is called the number of terminals, or the nodal strength. The tail of the distribution of the number of synaptic terminals, Figure 3(c), is adequately fit by a power law with exponent 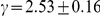).

Identifying neurons that play a central or special role in the transmission or processing of information may also prove useful [44]–[48]. To rank neurons according to their roles, we introduce several centrality indices. Perhaps the simplest centrality index is *degree centrality* 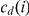. Degree centrality is simply the degree of a neuron, 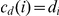, and is motivated by the idea that a neuron with connections to many other neurons has a more important or more central role in the network than a neuron connected to only a few other neurons. Neurons that have unusually high degree centrality include AVAL/R and AVBL/R. The same neurons lie in the tail of the distribution of the number of synaptic terminals, Figure 3(c), suggesting strong electrical coupling to the network. These neuron pairs are command interneurons responsible for coordinating backward and forward locomotion, respectively [22], [32], [49]. The high degree centralities of RIBL/R suggest a similarly central function for those neurons, though they each only have 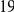 gap junction terminals, in the middle of the distribution of number of terminals, suggesting weaker electrical coupling to the network.

#### Small world properties

Having described statistical properties of individual neurons and connections, such as the degree and multiplicity distributions, we now investigate properties that may describe the efficiency of signal transmission across the gap junction network. Traditionally [14], this analysis does not consider multiplicity of gap junctions but treats them as binary. We analyze signal propagation when including multiplicities in the next subsection.

The geodesic distance, 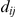, between two neurons in the network is the length of the shortest network path between them. The network path is measured by the number of connections that are crossed rather than by physical distance. The average geodesic distance over all pairs of neurons is the characteristic path length [14]:
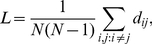
(2)where 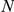 is the number of neurons. This global measure describes how readily or rapidly a signal can travel from one neuron to another since it is simply the average distance between all neurons. Clearly, the measure 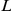 requires the network to be connected (otherwise 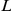 diverges), so we restrict attention to the giant component.

A signal originating in one neuron in the giant component must cross 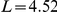 gap junction connections on average to reach another neuron of the giant component. For an Erdös-Rényi random network with 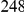 neurons and 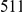 connections we computed the characteristic path length to be 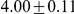 (Monte Carlo with 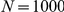). When the actual degree distribution of the gap junction network is taken into account, a random network from that ensemble would be expected to have characteristic path length 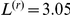 (see Methods). The distribution of geodesic distances 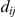 in the giant component is shown in Figure 1(a) in Text S4.

A second measure for signal propagation is the clustering coefficient 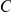, which measures the density of connections among an average neuron's neighbors. It is defined as [14]:
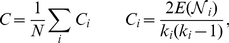
(3)where 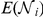 is the number of connections between neighbors of 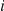, 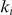 is the number of neighbors of 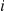, and 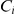 measures the density of connections in the neighborhood of neuron 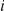 (we set 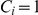 when 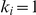). We find the clustering coefficient 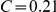. We computed the clustering coefficient for an Erdös-Rényi random network with 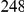 neurons and 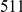 connections to be 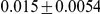 (Monte Carlo with 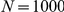). For a degree-matched random network, we computed the clustering coefficient 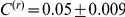 (Monte Carlo with 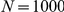). Thus, the giant component of the gap junction network is strongly clustered relative to random networks, both Erdös-Rényi and degree-matched.

Small world networks have much higher clustering coefficient relative to random networks without sacrificing the mean path length. For the giant component of the gap junction network, the corresponding ratios are 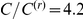 and 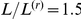, indicating that the network is small world. Quantitatively, small-world-ness of a network may be defined relative to a degree-matched Erdös-Rényi random network as follows [50]:
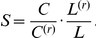
In the case of the giant component of the gap junction network, small-world-ness 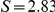.

Next we consider how quickly individual neurons reach all other neurons in the network. The normalized closeness of a neuron 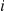 is the average geodesic distance 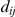 across all neurons 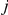 that are reachable from 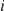 [45]:
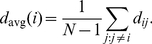
(4)The normalized closeness centrality, which takes higher values for more central neurons, is defined as the inverse, 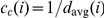.

Restricting to the gap junction giant component, the six most central neurons are AVAL, AVBR, RIGL, AVBL, RIBL, and AVKL. In addition to command interneuron classes AVA and AVB, these include RIBL and RIGL, both ring interneurons, and AVKL, an interneuron in the ventral ganglion of the head. The set of closeness central neurons mostly overlaps with the set of degree central neurons. The correlation between the two centrality measures does not extend to peripheral neurons, as the Spearman rank correlation coefficient [51] between degree centrality 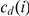 and closeness centrality 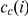 for the entire giant component is only 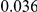.

#### Spectral properties

Global network properties discussed in the previous section characterize signal transmission while ignoring connection weights. As weights affect the effectiveness of signal transmission and vary among connections, we now analyze the weighted network by using linear systems theory. Although neuronal dynamics can be nonlinear, spectral properties nevertheless provide important insights into function. For example, the initial success of the Google search engine is largely attributed to linear spectral analysis of the World Wide Web [52].

We characterize the dynamics of the gap junction network by the following system of linear differential equations, which follow from charge conservation [53], [54].
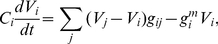
(5)where 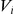 is the membrane potential of neuron 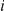, 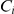 is the membrane capacitance of neuron 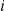, 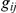 is the conductance of gap junctions between neurons 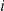 and 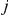, and 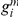 is the membrane conductance of neuron 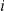. Assuming that each neuron has the same capacitance 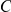 and each gap junction has the same conductance 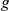, i.e. 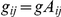, we can rewrite this equation in terms of the time constant 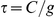 as:
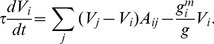
(6)


Assuming that gap junction conductance is greater than the membrane conductance, we temporarily neglect the last term and rewrite this equation in matrix form:
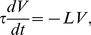
(7)where 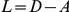 is the Laplacian matrix of the weighted network, 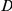 contains the number of neuron gap junctions on the diagonal and zeros elsewhere, and 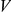 is a column vector of the membrane potentials.

**Figure 4:**
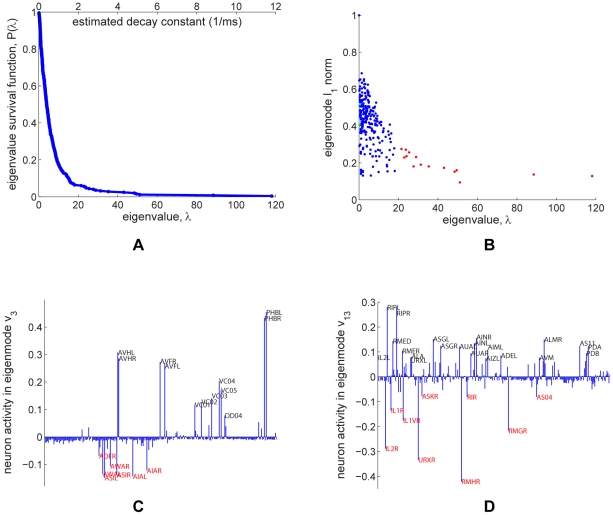
Linear systems analysis of the giant component of the gap junction network. (a). Survival function of the eigenvalue spectrum (blue). The algebraic connectivity, 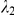, is 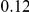 and the spectral radius, 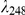, is 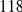. A time scale associated with the decay constant is also given. (b). Scatterplot showing the 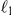 norm and decay constant of the eigenmodes of the Laplacian. The fastest modes from Figure 3 in Text S4 are marked in red. The sparsest and slowest modes, most amenable to biological analysis, are located in the lower-left corner of the diagram. (c). Eigenmode of Laplacian corresponding to 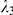 (marked green in panel (b)). (d). Eigenmode of Laplacian corresponding to 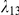 (marked cyan in panel (b)).

The system of coupled linear differential equations (6) can be solved by performing a coordinate transformation to the Laplacian eigenmodes. Since the Laplacian eigenmodes are decoupled and evolve independently in time, performing an eigendecomposition of initial conditions leads to a full description of the system dynamics. The survival function of the Laplacian eigenspectrum is shown in Figure 4(a).

What insight can be gained from inspection of the Laplacian eigenmodes? The gap junction network is equivalent to a network of resistors, where each gap junction acts as a resistor. The eigenmodes give intuition about experiments where a charge is distributed among neurons of the network and the spreading charge among the neurons is monitored in time. If the charge is distributed among neurons according to an eigenmode, the relative shape of the distribution does not change in time. The charge magnitude decays with a time constant specified by the eigenvalue. The smallest eigenvalue of the Laplacian is always zero, corresponding to the infinite relaxation time. In the corresponding eigenmode each neuron is charged equally.

If the charge is distributed according to eigenmodes corresponding to small eigenvalues, the decay is rather slow. Thus, these eigenmodes correspond to long-lived excitation. The existence of slowly decaying modes often indicates that the network contains weakly coupled subnetworks, in which neurons are strongly coupled among themselves. The corresponding charge distribution usually has negative values on one subnetwork and positive values on the other subnetwork. Because of the relatively slow equilibration of charge between the subnetworks, such an eigenmode decays slowly.

As an example of slow equilibration implying a subnetwork that is strongly internally coupled, one might speculate that the eigenmode associated with 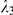 (Figure 4(c)) on the ‘black’ side reflects a coupling of chemosensory neurons in the tail (PHBL/R) along with interneurons (AVHL/R, AVFL/R) and motor neurons (VC01–05) involved in egg-laying behavior. At the level of gap junctions, these neurons are weakly coupled with chemosensory neurons in the head (ADFR, ASIL/R, AWAL/R) and related interneurons (AIAL/R) on the ‘red’ side.

Another interesting example is the eigenmode associated with 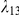 (Figure 4(d)). Neurons on the ‘red’ side overlap significantly with those identified previously in a hub-and-spoke circuit mediating pheromone attraction, oxygen sensing, and social behavior [55]. Such overlap is consistent with the view [55] that this network of neurons solves a consensus problem [56].

These two examples demonstrate that spectral analysis can uncover circuits that have been described using experimental studies. The probability of a known functional circuit appearing in an eigenmode by chance is small (see Methods). It would be interesting to see whether other eigenmodes have a biological interpretation and therefore generate predictions for future experiments.

To prioritize further analysis of eigenmodes for biological significance, it may be advantageous to focus on the slow and sparse modes, where few neurons exhibit significant activity. We can quantify sparseness of normalized eigenmodes by the sum of absolute values of the eigenmode components, also known as the 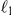 norm or Manhattan distance. Sparser eigenmodes have smaller 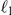 norms [57]. Figure 4(b) is a scatterplot of eigenmodes showing both their decay constant and their 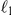 norms.

The full set of eigenmodes of the connected component is shown in Figure 2 in Text S4. The eigenmodes corresponding to large eigenvalues decay fast, suggesting that corresponding neurons have the same membrane potential on relevant time scales and act effectively as a single unit. Many such eigenmodes peak (with opposite signs) for left-right neuronal pairs (Figure 3 in Text S4), often known to be functionally identical, which therefore act as a single unit.

To understand timescales, one might wonder what the absolute values of decay constants for various eigenmodes are. Current knowledge of electrical parameters for *C. elegans* neurons allows us to estimate the decay times only approximately. Assuming neuron capacitance of 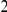
pF [58] and gap junction conductance of 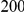
pS, we find a time constant 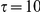
ms. This implies that the slowest non-trivial mode corresponding to the second lowest eigenvalue, 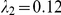 has decay time of about 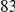
ms, Figure 4(a). This eigenvalue, 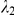, is known as the algebraic connectivity of a network [59].

What is the effect of the dropped term corresponding to the membrane current in (6)? As this term would correspond to adding a scaled identity matrix to the Laplacian, the spectrum should uniformly shift to higher values by the corresponding amount. Thus, even the eigenmode corresponding to the zero eigenvalue would now have a finite decay time. Assuming the membrane conductance of about 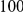
pS [58], we find 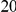
ms decay time. This leads to a 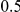 increase in the values of 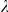. Now, the slowest non-trivial mode corresponds to a decay time of about 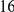
ms.

In addition to highlighting groups of neurons that could be functionally related, spectral analysis allows us to predict, under linear approximation, the outcome of experiments that study the spread of an arbitrarily generated excitation in the neuronal network. Such excitation can be generated in sensory neurons by presenting a sensory stimulus [60] or in any neuron by expressing a light-gated ion channel, such as channelrhodopsin, in that cell and stimulating optically [26], [61], [62]. The spread of activity can be monitored electrophysiologically or using calcium-sensitive indicators.

To predict the spread of activity, we may decompose the excitation pattern into the eigenmodes and, by taking advantage of eigenmode independence, express temporal evolution as a superposition of the independently decaying eigenmodes. The initial redistribution of charge would correspond to the fast eigenmodes, whereas the long-term evolution of charge distribution would be described by the slow eigenmodes. Text S3 further discusses eigendecomposition and the interpretation of eigenmodes.

Understanding propagation of neuronal activity in response to stimulation (either for the complete network or for ablation studies) may also be carried out directly in the time domain by stepping through the dynamics in (6) or more electrophysiologically realistic nonlinear dynamics. Predictions of experimental results would then be determined by stimulating and measuring exactly as in the experiment itself.

#### Motifs

**Figure 5:**
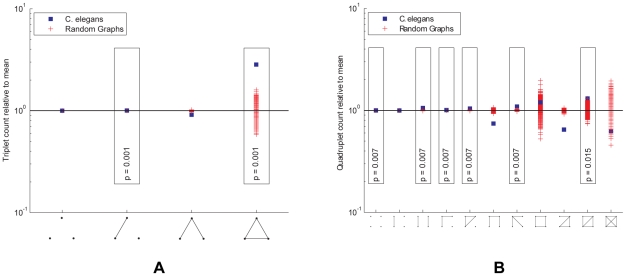
Subnetwork distributions for the gap junction network. Overrepresented subnetworks are boxed, with the 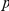-value from the step-down min-P-based algorithm for multiple-hypothesis correction [64], [77] (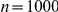) shown inside. (a). The ratio of the 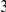-subnetwork distribution and for the mean of a degree-preserving ensemble of random networks (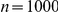). The counts for the particular random networks that appeared in the ensemble are also shown. (b). The ratio of the 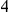-subnetwork distribution and for the mean of a degree and triangle-preserving ensemble of random networks (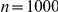). The counts for the particular random networks that appeared in the ensemble are also shown.

Several of the quantitative properties computed thus far measure global network structure or individual neuron properties. Now we analyze the frequency of various connectivity subnetworks among small local groups of neurons. Overrepresentation in the subnetwork distribution often displays building blocks of the network such as computational units [17], [63]. Since the gap junction network is undirected, there are four kinds of subnetworks that can appear over three neurons; this distribution is shown in Figure 5(a). As a null-hypothesis we use random network ensembles that preserve the degree distribution. We find that fully connected triplets are overrepresented.

Four neurons can be wired into 11 kinds of subnetworks; this distribution is shown in Figure 5(b). In the case of quadruplets, the null-hypothesis preserves the degree for each neuron and the number of triangles. A numerical rewiring procedure is used to generate samples from these random network ensembles [38], [64], since no analytical expression for expected subnetwork counts is extant [65]. We find that a “fan” (motif #7) and a “diamond” (motif #10) are overrepresented.

Note that neurons participating in motifs also make connections with neurons outside of the motif, which are traditionally not drawn in putative functional circuits [8], [60]. Such putative functional circuit diagrams may even omit connections within the motif [8], [60], which we do not allow.

### Chemical Synapse Network

Now we consider the chemical synapse network. Recall that due to structural differences between presynaptic and postsynaptic ends of a chemical synapse, electron micrographs can be used to determine the directionality of connections. Hence the adjacency matrix is not symmetric as it was for the gap junction network.

#### Basic structure and connectivity

The network that we analyze consists of 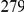 neurons and 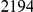 directed connections implemented by one or morhemical synapses. The adjacency matrix of the network shown in Figure 1 is suggestive of a three-layer architecture. The distribution of connections between categories, Table 3 in Text S4, reveals that each chemical subnetwork is characterized by a high number of recurrent connections, just as for the gap junction. However, the majority of connections with other subnetworks is consistent with feedforward information processing (sensory to interneuron and interneuron to motorneurons). Therefore, a three-layer network abstraction may be more valuable for chemical synapses than for gap junctions.

There are two different definitions of connectivity for directed networks. A weakly connected component is a maximal group of neurons which are mutually reachable by possibly violating the connection directions, whereas a strongly connected component is a maximal group of neurons that are mutually reachable without violating the connection directions. The whole chemical synapse network is weakly connected and can be divided into a giant strongly connected component with 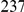 neurons, a smaller strongly connected component of 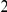 neurons, and 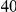 neurons that are not strongly connected (Table 4 in Text S4).

The random directed network corresponding to the chemical network is fully weakly connected, even when the degree distribution is taken into account (see Methods). A strongly connected giant component as small as in the chemical network is not likely in a random network (see [66]). Thus, the chemical network is more segregated than would be expected for a random network.

#### Distributions of degree, multiplicity and the number of terminals

**Figure 6:**
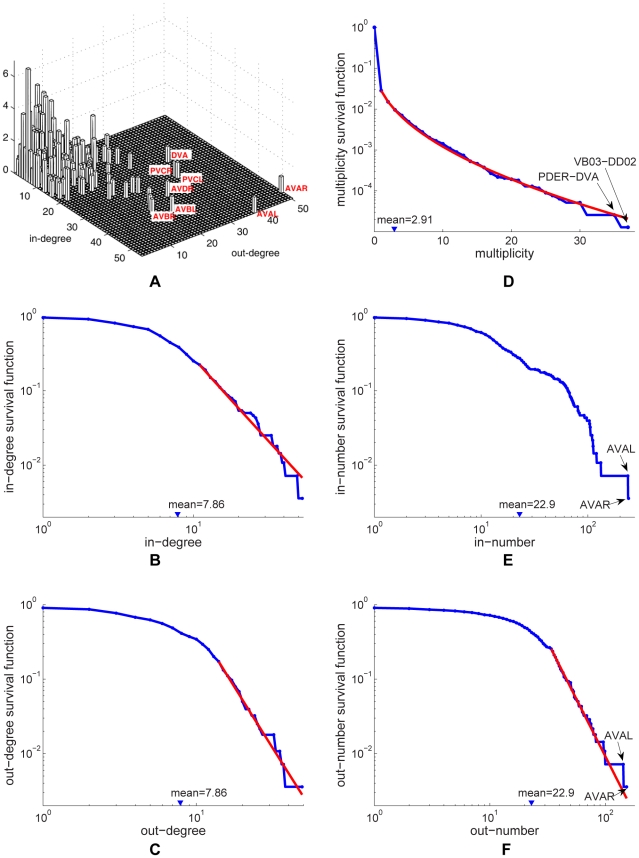
Degree distribution (a) and survival functions for the distributions of in-/out-degree, multiplicity, and in-/out-number of synaptic terminals in the chemical synapse network (b)–(f). Neurons or connections with unusually high statistics are labeled. The tails of the distributions can be fit by a power law with exponents 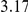 for in-degree (b); 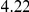 for out-degree (c); and 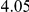 for out-number (f). The exponents for the survival function fits can be obtained by subtracting one. The survival function of the multiplicity distribution for 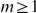 can be fit by a stretched exponential of the form 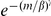 where 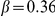 and 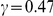 (d). No satisfactory fit was found for the distribution of in-numbers (e).

Since chemical synapses form a directed network, neuron connectivity is characterized by in-degrees (the number of incoming connections) and out-degrees (the number of outgoing connections) rather than simply degrees. The joint distribution of in-degrees and out-degrees is shown in Figure 6(a). As can be seen by the distribution clustering around the diagonal line, the in-degrees and out-degrees are correlated; the Pearson correlation coefficient is 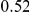 (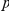-value 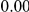), very similar to the Pearson correlation coefficient of an email network, 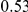, though the email network was much larger (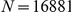) [67].

The survival functions associated with the marginal distributions of in-degrees and out-degrees are shown in Figures 6(b) and 6(c) respectively. The mean number of incoming and outgoing connections is 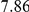 each. We attempt to fit these distributions. The tails of the two distributions can be satisfactorily fit by power laws with exponents 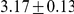 and 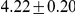 respectively. Exponential fit is ruled out (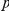-value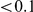) for the in-degree but not for the out-degree distribution. In the latter case, the log-likelihood is insignificantly lower for the exponential decay than for the power law.

Multiplicity of connection, 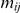, is the number of synapses in parallel from neuron 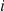 to neuron 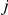. The corresponding survival function (including unconnected pairs) is shown in Figure 6(d). The mean number of synapses per connection (excluding unconnected pairs) is 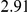. The tail of the distribution can be fitted by an exponential, but not by a power law (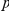-value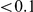). In addition, the whole distribution (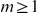) can be fit by a stretched exponential (or Weibull) distribution, 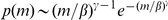 with the scale parameter 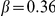 and the shape parameter 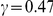. A stretched exponential applied to the whole distribution has the same number of fitting parameters as an exponential decay fit to the tail starting with an adjustable 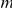. Log-likelihood comparison of the tail exponential and the stretched exponential favors the latter.

As for the gap junction network, we can also study the distribution of number of synaptic terminals on a neuron. This involves adding the multiplicities of the connections, rather than just counting the number of pre- or post-synaptic partners. The joint histogram (not shown) exhibits similar correlation as for the degree distribution, with Pearson correlation coefficient 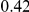 (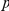-value 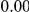).

Figures 6(e) and 56(f) show the marginal survival functions for the number of post-synaptic terminals (in-number) and the number of pre-synaptic terminals (out-number). The mean number of pre- and post-synaptic terminals is 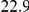 each. We were unable to find a satisfactory simple fit to the in-number distribution, Figure 6(e). The tail of the out-number distribution could be fit by a power law with exponent 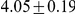, but not by an exponential, Figure 6(f).

As for the gap junction network, we can identify central neurons (cf. [47], [68]) for the chemical network. The degree centrality in a directed network may be defined with respect to the in-degree or the out-degree. Interestingly, neuron AVAL has the largest in-degree and AVAR has the second largest in-degree, whereas AVAR has the largest out-degree and AVAL has the second largest out-degree, Figure 6(a).

#### Small world properties

In the strongly connected component, we can define the directed geodesic distance as the shortest path between two neurons that respects the direction of the connections. The distribution of the directed geodesic distance, Figure 1(b) in Text S4, is characterized by the mean path length, 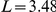 computed over all pairs of neurons in the strongly connected component. For a random network degree-matched to the chemical network, one would expect 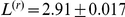 (Monte Carlo with 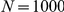) and the ratio 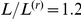.

Although there are several definitions of clustering for directed graphs in the literature [69], we use the clustering of the out-connected neighbors since it captures signal flow emanating from a given neuron:
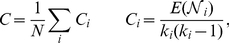
(8)where 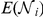 is the number of connections between out-neighbors of neuron 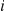, 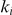 is the number of out-neighbors of 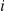, and 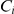 measures the density of connections in the neighborhood of neuron 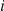. For the chemical network, the clustering coefficient is 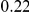. For a degree-matched random network we computed the clustering coefficient 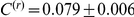 (Monte Carlo with 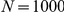) and the ratio 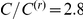. Therefore, the chemical strongly connected component is a small-world network with 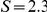.

For directed networks, measures of in-closeness and out-closeness may be defined using the average directed geodesic distance. In particular, the normalized in-closeness is the average geodesic distance from all other neurons to a given neuron:
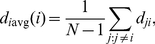
(9)and the out-closeness is the average geodesic distance from a given neuron to all other neurons:
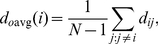
(10)where 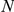 is the number of neurons. Normalized centralities are the inverses: 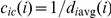 and 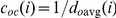. The motivation behind these indices is similar to that in the gap junction case. In-closeness central neurons can be easily reached from all other neurons in the network. Out-closeness central neurons can easily reach all other neurons in the network. Normalized in-closeness centrality 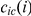 and normalized out-closeness centrality 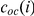 are weakly anti-correlated, with Pearson correlation coefficient 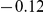 (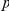-value 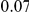).

For the giant component of the chemical network, the most in-closeness central neurons include AVAL, AVAR, AVBR, AVEL, AVER, and AVBL. All are command interneurons involved in the locomotory circuit; these neurons are also central in the gap junction network. The in-closeness centrality of command interneurons may indicate that in the *C. elegans* nervous system, signals can propagate efficiently from various sources towards these neurons and that they are in a good position to integrate it.

The most out-closeness central neurons include DVA, ADEL, ADER, PVPR, AVJL, HSNR, PVCL, and BDUR. Only PVCL is a command interneuron involved in locomotion. The neuron DVA is an interneuron that performs mechanosensory integration; ADEL/R are sensory dopaminergic neurons in the head; and the other central neurons are interneurons in several parts of the worm. The out-closeness centrality of these neurons may indicate that signals can propagate efficiently from these neurons to the rest of the network and that they are in a good position for broadcast.

#### Spectral properties

Although chemical synapses are likely to introduce more nonlinearities than gap junctions, linear systems analysis can provide interesting insights, especially in the absence of other tools. Such an approach has additional merit in *C. elegans*, where neurons do not fire classical action potentials [58] and have chemical synapses that likely release neurotransmitters tonically [54]. To justify such analysis, a system of linear equations may be derived by approximating sigmoidal synaptic transmission functions with a Taylor series expansion around an equilibrium point [54].

A major source of uncertainty in linear systems analysis of the chemical network is the unknown sign of connections, i.e. excitatory or inhibitory, due to the difficulty in performing electrophysiology experiments. We use a rough approximation that GABAergic neurons make inhibitory synapses, whereas glutamergic and cholinergic neurons form excitatory synapses [70], but see [60]. The following 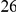 neurons express GABA [71]: DVB, AVL, RIS, DD01–DD06, VD01–VD13, and the four RME neurons.

Similarly to the gap junction network, we write the system of linear differential equations for the chemical synapse network [53], [54]:
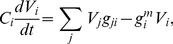
(11)where 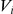 is the membrane potential of neuron 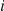 measured relative to the equilibrium, 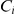 is the membrane capacitance of neuron 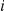, 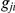 is the conductance in neuron 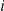 contributed by a chemical synapse in response to voltage 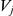 measured relative to the equilibrium and 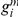 is the membrane conductance of neuron 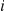. Assuming that each neuron has the same capacitance 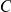 and each chemical synapse contact has the same conductance 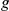, i.e. 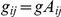, we can rewrite this equation in terms of the time constant 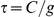 as:
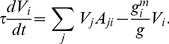
(12)


To avoid redundancy we defer analyzing this system of differential equations to the next section, where we consider the combined system including both gap junctions and chemical synapses.

#### Motifs

We also find subnetwork distributions for the chemical synapse network. Since the network is directed, there are many more possible subnetworks. In particular there are 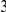 possible subnetworks on two neurons and 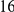 possible subnetworks on three neurons. We identify overrepresented subnetworks by comparing to random networks, generated with a rewiring procedure [38], [64]. Such random network ensembles preserve in-degree and out-degree in the case of doublets and, additionally, the numbers of bidirectional and unidirectional connections for each neuron in the case of triplets.

**Figure 7:**
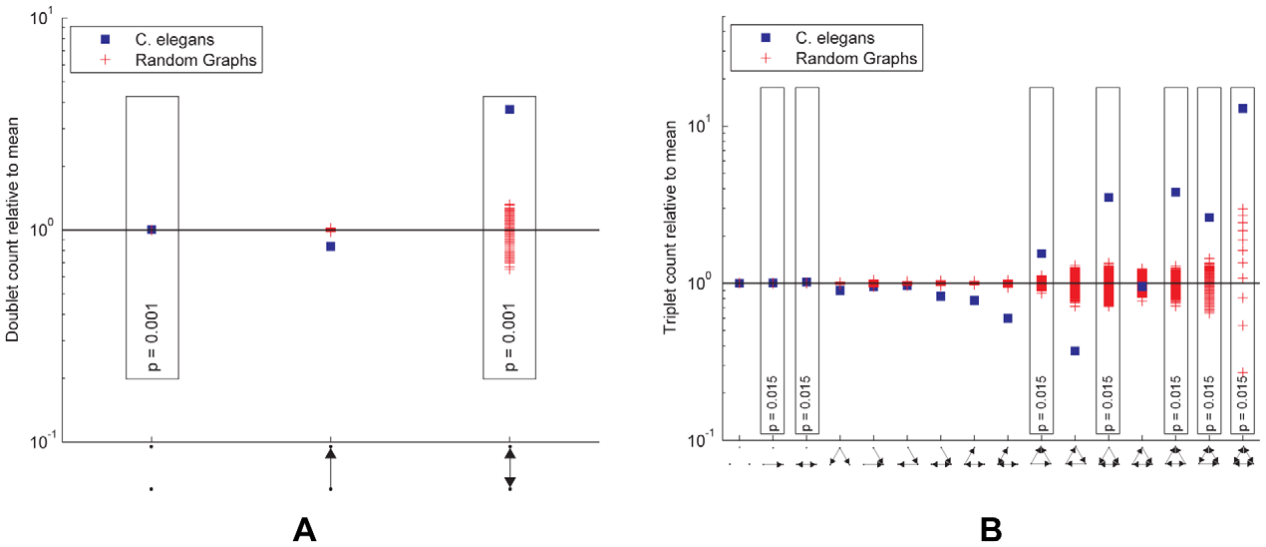
Subnetwork distributions for the chemical synapse network. Overrepresented subnetworks are boxed, with the 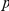-value from the step-down min-P-based algorithm for multiple-hypothesis correction [64], [77] (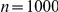) shown inside. (a). The ratio of the 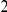-subnetwork distribution and the mean of a random network ensemble (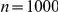). Realizations of the random network ensemble are also shown. (b). The ratio of the 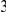-subnetwork distribution and the mean of a random network ensemble (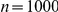). Realizations of the random network ensemble are also shown.

Figures 7(a) and 7(b) show the subnetwork distributions on two and three neurons, respectively. We find that the *C. elegans* network contains similar overrepresented subnetworks as found by analyzing incomplete data [17], [64]. For example, there is greater reciprocity in the chemical network than would be expected in a random network. Similarly, triplets with connections (of any direction) between each pair of neurons (seven rightmost triplets in Figure 7(b)) collectively occur with much greater frequency than would be expected for a random network.

Overrepresentation of reciprocal [8, Ch. 7] and triangle [7] motifs were previously noted. Such overrepresentation would arise naturally if proximity was a limiting factor for connectivity, however there is no evidence for this limitation. Thus, motifs may have a functional role.

### Full Network

Having considered the gap junction network and the chemical synapse network separately, we also examine the two networks collectively. To study the two networks, one may either look at a single network that takes the union of the connections of the two networks or one may look at the interaction between the two networks.

#### Single combined network

First we look at a combined network, which is produced by simply adding the adjacency matrices of the gap junction and chemical networks together, while ignoring connection weights. Thus we implicitly treat gap junction connections as double-sided directed connections. This new network consists of 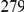 neurons and 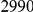 directed connections. It has one large strongly connected component of 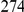 neurons and 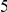 strongly isolated neurons. The five isolated neurons are IL2DL/R, PLNR, DD06, and PVDR; this set is simply the intersection of the isolated neurons in the gap junction and chemical networks and does not seem to have any commonalities among members. Of course, it follows that since the chemical network is a single weakly connected component, this combined network is also a single weakly connected component.

Naturally, the combined network is more compact than the individual networks. The mean path length 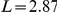, the geodesic distance distribution (Figure 1(c) in Text S4) becomes narrower. For a random network degree-matched to the combined network, one would expect 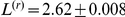 (Monte Carlo with 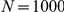) and the ratio 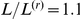. The clustering coefficient for the combined network is 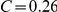. The clustering coefficient for a degree-matched random network 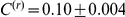 (Monte Carlo with 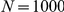) and the ratio 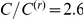. Therefore, the combined network is small-world, just like individual networks, with 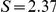.

Turning to closeness centrality, the most in-close central neurons are AVAL/R, AVBR/L, and AVEL/R, as would be expected from the individual networks. The most out-close central neurons are DVA, ADEL, AVAR, AVBL, and AVAL, which include the top out-close neurons for both individual networks.

We can also calculate the degree distribution of this combined network. The Pearson correlation coefficient between the in-degree and out-degree is 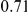 (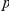-value 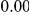); it is not surprising that the coefficient is so large considering that the gap junctions introduce an in- and out-connection simultaneously. Similar to the chemical synapse network, the tails of both the in-degree and the out-degree survival functions (Figures 4(a) and 4(b) in Text S4) can be fit with power laws. The tail of the out-degree could also be fit by an exponential decay, albeit with lower likelihood.

The neurons with the greatest degree centrality are AVAL and AVAR. As for the chemical synapse network, neuron AVAL has the largest in-degree and AVAR has the second largest in-degree, whereas AVAR has the largest out-degree and AVAL has the second largest out-degree (Figures 4(a) and 4(b) in Text S4). The next two neurons are AVBL/R in both in-degree and out-degree senses.

As for the chemical synapse network, the tail of the out-number distribution was fit by a power law and the tail of the in-number distribution could not be fit satisfactorily. The tail of the out-number distribution could also be fit by an exponential, albeit with lower likelihood. The multiplicity can be fit satisfactorily by a stretched exponential.

#### Spectral properties

In this section we apply linear systems analysis to the combined network of chemical synapses and gap junctions taking into account multiplicities of individual connections. Due to our ignorance about the relative conductance of a single gap junction and of a single chemical synapse, we assume that they are equal. By combining equations (6) and (12) we arrive at:
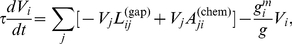
(13)where 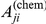 is negative if neuron 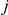 is GABAergic and positive otherwise.

**Figure 8:**
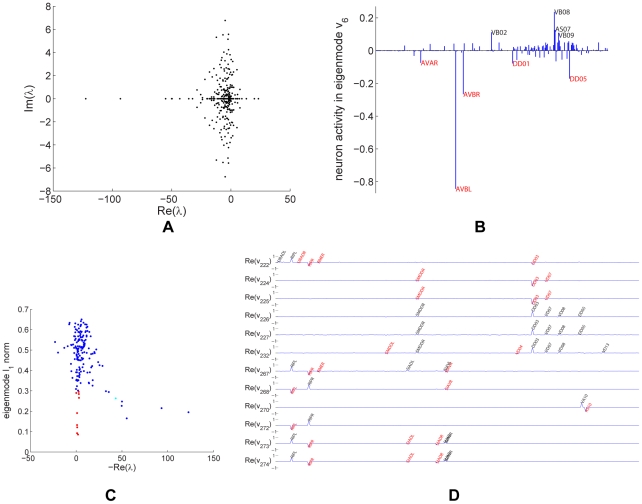
Linear systems analysis for the strong giant component of the combined network. (a). Eigenvalues plotted in the complex plane. (b). The eigenmode associated with eigenvalue 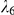 (marked cyan in panel (c)). (c). Scatterplot showing the sparseness and decay constant of the eigenmodes. (d). Sparse and slow eigenmodes of the combined network (marked red in panel (c)). The real parts of the eigenmodes corresponding to 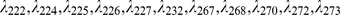, and 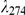 are shown. The eigenmodes are labeled with neurons that take value above a fixed absolute value threshold. Neurons with negative values are in red, whereas neurons with positive values are in black.

We proceed to find a spectral decomposition for the combined network. To avoid trivial eigenmodes, we restrict our attention to the strongly connected component of the combined network containing 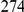 neurons. As before, we ignore the 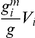 term and only study the matrix 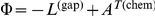. Since 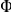 is not symmetric, eigenvalues and eigenmodes may be complex-valued, occurring in complex conjugate pairs. Eigenvalues are plotted in the complex plane in Figure 8(a).

What is the meaning of complex eigenvalues? The imaginary part of an eigenvalue is the frequency at which the associated eigenmode oscillates. The real part of an eigenvalue determines the amplitude of the oscillation as it varies with time. Eigenmodes that have an eigenvalue with a negative real part decay with time, whereas eigenmodes that have an eigenvalue with a positive real part grow with time. When examining the temporal evolution of the eigenmodes whose eigenvalues are shown in Figure 8(a), one should keep in mind that the ignored 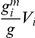 term would shift the real part of the eigenvalues towards more negative values.

As for the gap junction network alone, we can look for eigenmodes that may have functional significance. For example, the sixth eigenmode of the combined network, Figure 8(b), includes neurons that are involved in sinusoidal body movement. As before, one may focus on sparse and slow eigenmodes for ease of investigation. The distribution of 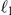 norm and real part of eigenvalues is shown in Figure 8(c), and twelve of the sparsest and slowest eigenmodes of the combined network are plotted in Figure 8(d).

Having the eigenspectrum of the combined network allows one to calculate the response of the network to various perturbations. By decomposing sensory stimulation among the eigenmodes and following the evolution of each eigenmode, one could predict the worm's response to the stimulation. A similar calculation could be done for artificial stimulation of the neuronal network, induced for example, using channelrhodopsin [26], [61], [62].

As noted for the gap junction alone, the network may also be studied in the time domain directly by stepping through the dynamics in (13) or more electrophysiologically realistic nonlinear dynamics.

#### Interaction between networks

We have measured the structural properties of the combined network formed by adding together the adjacency matrices of the gap junction and chemical synapse network, however it is unclear how they interact. The two networks could be independent, or their connections could overlap more or less often than by chance.

**Figure 9:**
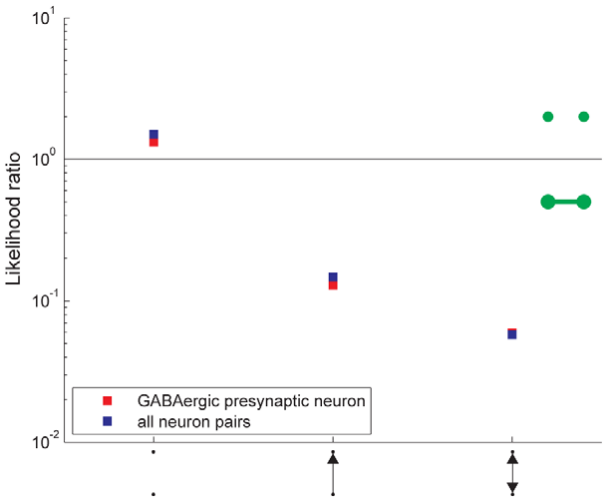
Likelihood ratio for the possible chemical network doublets (horizontal axis) given the absence/presence of a gap junction between the two neurons (as indicated by the green marks).

To investigate how the two networks overlap, we look at local structure. Figure 9 shows the likelihood ratios of chemical synapse connections being absent, being unidirectional, and being bidirectional given the presence or absence of a gap junction between the same pair of neurons (see Methods). As can be seen, chemical synapses are more likely to be absent when there is no gap junction than when there is one. Unidirectional, and especially bidirectional, chemical synapses are more likely when there is a gap junction between given neurons. In this sense, the two networks are correlated, however it should be noted that when there is a gap junction, about 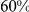 of the time there is no chemical synapse.

Durbin had found that chemical and gap junction networks are essentially independent when imposing physical adjacency restrictions [8, Ch.∼7], but as noted above, there is no evidence that proximity is a limiting factor for connectivity in *C. elegans*. Thus, there may be a functional role for correlation/anticorrelation of the joint presence of gap junction and chemical connections.

Why might the presence of connections in two networks either be correlated or anticorrelated? One possibility is that correlated connections simultaneously perform different functions [72] whereas anticorrelation yields connections between distinct kinds of neuronal pairs [73], [74], [Tavoularis CR and Wicker SB, unpublished].

What are the different functions performed by chemical synapses and gap junctions that could lead to correlation? One possibility is that the two different functions are sign-inverting and non-inverting coupling. Gap junctions are non-inverting: higher potential in a neuron raises the potential in other gap-junction-coupled neurons. Chemical synapses, on the other hand, may be either excitatory (non-inverting) or inhibitory (inverting). When the likelihood computations are repeated considering only neuron pairs where the presynaptic neuron is known to be GABAergic [71], there is not much change, see Figure 9. This suggests that the primary purpose of overlapping inhibitory chemical synapses is not to counter excitatory gap junctions. Some other reason, such as differing temporal properties or robustness from redundancy, is needed to explain correlation. This result, however, is only suggestive since the neurotransmitters and their action on postsynaptic receptors in many neurons have not been determined.

Another measure of the interaction between the two networks is the correlation between the degree sequences. The Pearson correlation coefficient between the gap junction degree and the chemical network in-degree is greater than the Pearson correlation coefficient between the chemical network in-degree and out-degree. The Pearson correlation coefficient between the gap junction degree and the chemical network out-degree is less than the Pearson correlation coefficient between the chemical network in-degree and out-degree. This is shown in Table 1 where comparisons to Pearson correlation coefficients between randomly permuted degree sequences (see Methods) are also shown. Large Pearson correlation coefficients imply that neurons are ordered in similar ways according to degree centrality.

**Table 1:** Degree sequence correlation Coefficients.

	gap/in	gap/out	in/out	email [67]
correlation coefficient 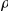 ( 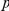 -value)	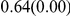	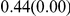	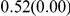	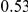
avg. rand. perm. 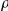	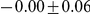	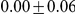	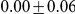	

The two networks seem to primarily reinforce each other with correlated structure rather than augment each other with anticorrelated connections.

### Robustness Analysis

Although the reported wiring diagram corrects errors in previous work and is considered self-consistent, one might wonder how remaining ambiguities and errors in the wiring diagram might affect the quantitative results presented. Furthermore there are connectivity pattern differences among individual worms; these individual variations may have similar effects on the analysis as errors and ambiguities.

For network properties that are defined locally, such as degree, multiplicity, and subnetwork distributions, clearly small errors in the measured wiring diagram lead to small errors in the calculated properties. For global properties such as characteristic path length and eigenmodes, things are less clear.

To study the robustness of global network properties to errors in the wiring diagram, we recalculate these properties in the wiring diagrams with simulated errors. We simulate errors by removing randomly chosen synaptic contacts with a certain probability and assigning them to a randomly chosen pair of neurons. Then, we calculate the global network properties on the ensemble of edited wiring diagrams. The variation of the properties in the ensemble gives us an idea of robustness.

First, we explore the robustness of the small world properties and the giant component calculations. We edit wiring diagrams by moving each gap junction contact with 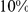 probability and chemical synapse contact with 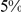 probability. Tables 5 and 6 in Text S4 show the global properties for 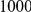 random networks obtained by editing the experimentally measured network. These tables suggest that our quantitative results are reasonably robust to ambiguities and errors in the wiring diagram.

Properties for the neuronal network from prior work in [13] are also shown for comparison. The number of synaptic contacts that must be moved to achieve this network (editing distance) roughly corresponds to that with 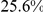 probability.

**Figure 10:**
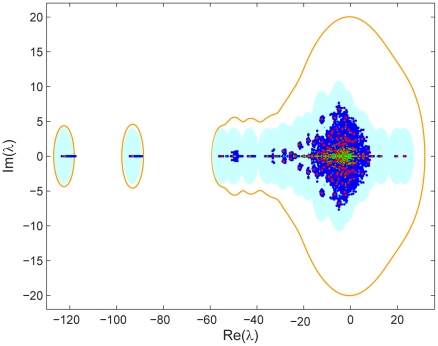
The spectrum of the giant component of the combined network matrix 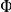 (red), ε-disks around the spectrum (light blue), spectra of 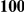 randomly edited networks (blue), and the ε-pseudospectrum (orange). The value 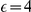 is used (the average spectral norm of the 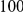 editing matrices was 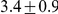). The spectrum of the giant component of the combined network matrix 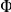 under an alternate quantitation of send_joint synapses is also shown (green).

Second, we characterize robustness for the linear systems analysis. Because of greater sensitivity of the eigenvalues to errors, we edit wiring diagrams by moving each gap junction contact with 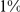 probability and a chemical synapse contact with 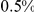 probability. The spectra for 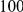 randomly edited networks along with the spectrum for the measured network (Figure 8(a)) are shown in Figure 10. Although the locations of eigenvalues shift in the complex plane, many of them move less than the nearest neighbor distance and remain isolated.

In addition to considering the effect of typical random edits, we can characterize the effect of worst-case errors on the eigenvalues using the 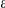-pseudospectrum [75], which gives the eigenvalue loci 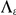 for all perturbations by matrices of norm 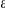 (Figure 10). For the gap junction, 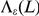 is simply the set of disks of radius 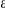 around the eigenvalues, but for the chemical and combined networks, 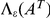 and 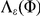 are larger. In the worst case scenario, most eigenmodes become mixed up.

Electron micrographs of chemical synapses have a further ambiguity when more than one postsynaptic partner receives input at a release site. We treated such polyadic (send_joint) synapses no differently than other synapses, but one might alternatively determine multiplicity by counting such synapses at 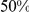 strength. This alternate quantitation clearly does not change statistics that ignore multiplicity; the change in the spectrum is depicted in Figure 10.

Small deviations from equality when weighting gap junctions and chemical synapses to form the combined network yield similar spectral changes as the alternate quantitation of chemical synapses displayed in Figure 10.

## Discussion

We have presented a corrected and more comprehensive version of the neuronal wiring diagram of hermaphrodite *C. elegans* using materials from White *et al.* [7] and new electron micrographs. Despite the significant additions, this wiring diagram is still incomplete due to methodological limitations discussed in the An Updated Wiring Diagram section. Yet, our work represents the most comprehensive mapping of the neuronal wiring diagram to date. The sensitivity of our analysis to methodological limitations (and to network structure variation among individual organisms) is discussed in the Robustness Analysis section.

We proposed a convenient way to visualize the neuronal wiring diagram. The corrected wiring diagram and its visualization should help in planning experiments, such as neuron ablation.

Next, we performed several statistical analyses of the corrected wiring, which should help with inferring function from structure.

By using several different centrality indices, we found central neurons, which may play a special role in information processing. In particular, command interneurons responsible for worm locomotion have high degree centrality in both chemical and gap junction networks. Interestingly, command interneurons are also central according to in-closeness, implying that they are in a good position to integrate signals. However, most command interneurons do not have highest out-closeness, meaning that other out-closeness central neurons, such as DVA, ADEL/R, PVPR, etc., are in a good position to deliver signals to the rest of the network.

Linear systems analysis yielded a principled methodology to hypothesize functional circuits and to predict the outcome of both sensory and artificial stimulation experiments. We have identified several modes that map onto previously identified behaviors.

Networks with similar statistical structural properties may share functional properties thus providing insight into the function of the *C. elegans* nervous system. To enable comparison of the *C. elegans* network with other natural and technological networks [76], we computed several structural properties of the neuronal network. In particular, the gap junction network, the chemical synapse network, and the combined neuronal network may all be classified as small world networks because they simultaneously have small average path lengths and large clustering coefficients [14].

The tails of the degree and terminal number distributions for the gap, chemical and combined networks (with the exception of the in-numbers) follow a power law consistent with the network being scale-free in the sense of Barabási and Albert [40]. The tails of some distributions can also be fit by an exponential decay, consistent with a previous report [15]. However, we found that exponential fits for the tails have (sometimes insignificantly) lower log-likelihoods than power laws, making the exponential decay a less likely alternative. For whole distributions, neither distribution passes the 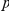-value test; if one is forced to choose, the exponential decay may be a less poor alternative.

Several statistical properties of the *C. elegans* network are similar to those of the mammalian cortex. In particular, the whole distribution of *C. elegans* chemical synapse multiplicity is well-fit by a stretched exponential (or Weibull) distribution (Figure 6(d)). Taking multiplicity as a proxy of synaptic connection strength, this is reminiscent of the synaptic strength distribution in mammalian cortex, which was measured electrophysiologically, [30], [77]. The definition of stretched exponential distribution is slightly different [30], but has the same tail behavior. The stretch factor is 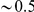, close to that in the cortical network.

In addition, we found that motif frequencies in the chemical synapse network are similar to those in the mammalian cortex [77]. Both reciprocally connected neuron pairs and triplets with a connection between every pair of neurons (regardless of direction) are over-represented. The similarity of the connection strength and the motif distributions may reflect similar constraints in the two networks. Since proximity is unlikely to be the limiting factor, we suggest that these constraints may reflect functionality. We found that the chemical synapse and the gap junction networks are correlated, which may provide insight into their relative roles.

To conclude the paper, let us note that our scientific development was not hypothesis-driven, but rather exploratory. Yet we hope that the reported statistics will help in formulating a theory that explains how function arises from structure.

## Materials and Methods

### Data Acquisition

This section describes the methods used to determine neuronal connectivity; see [78] for further details.

We started assembling the wiring diagram by consolidating existing data from both published and unpublished sources. Using J. G. White *et al.*'s The Mind of a Worm (MOW) [7] as the starting point, we extracted wiring data from diagrams, figures, tables, and text (for example, see [7, Appendix A, pp. 118–122] on neuron AVAL/R). The connectivity of each neuron, its synaptic partner, and synaptic type (chemical, gap junction, neuromuscular) was manually entered into an electronic database. In the ventral cord, determining this level of synaptic specification was complicated by the fact that connections were recorded by neuron class. For example, bilateral neurons PVCL and PVCR were simply listed as PVC. We assigned proper connections to the appropriate left/right neuron by referring to White and coworker's original laboratory notebooks and original electron micrographs. In some cases, the number of synapses for a given neuron class in MOW differed from the sum of connections for the bilateral pairs in the notebooks and/or electron micrographs. The synaptic value of these neurons was determined by taking the value in MOW and dividing it between the left/right neurons proportionally to the values in the notebooks and/or electron micrographs.

Next we incorporated R. M. Durbin's data for the anterior portion of the worm, reconstructed from animal *N2U* [8]. For neurons that projected beyond the nerve ring, only the anterior connections needed update. Since data from MOW did not specify the location of synapses, integration proved difficult. For these neurons, we obtained positional information by cross-referencing Durbin's data against original electron micrographs and his handwritten annotations in White's laboratory notebooks. Only synapses located in regions addressed by Durbin were included. Connections in the middle and tail regions of the worm were mostly unaffected by these updates.

Studies based on green fluorescent protein (GFP) reporters mostly confirm the electron micrograph reconstructions described in MOW. A few differences between GFP-stained neurons and White's work have been observed [Hobert O and Hall DH, unpublished]. Notably, the anterior processes of DVB and PVT could have been mistakenly switched in MOW [7]. Based on these findings, we reversed the connections for neurons DVB and PVT anterior to the vulva.

Most published works have focused on the neck and tail regions of *C. elegans* where most neuron cell bodies reside. Reconstructions of neurons in the mid-body of the worm, on the other hand, are scant and incomplete. From a combination of published works [7], [8], [10], [79], we found that wiring data for 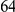 neurons had large gaps or were missing entirely. Sixty-one of these were motor neurons in the ventral cord. Two were excretory neurons (CANL/R) that do not appear to make any synapses. The remaining neuron, RID, is the only process in the dorsal cord that extends over the length of the animal.

At the *C. elegans* archive (Albert Einstein College of Medicine), we uncovered a large number of reconstruction records in White *et al.*'s laboratory notebooks. These notebooks identified neurons by different color code labels depending on the animal, the location of the neurite (ventral or dorsal), and magnification of the electron micrograph. To ascertain the identity of the neurons, we relied on a combination of color code tables and comparisons of common anatomical structures between electron micrograph prints. In the end, we identified notes for full reconstructions of 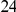 of the aforementioned neurons. Partial connectivity data for the remaining 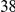 were also available where 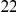 neurons have partial/missing dorsal side connections and 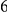 neurons have partial ventral side connections. We checked the connections of all (both published and unpublished) neurons in the ventral cord against electron micrographs used by White and coworkers. Over 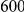 updates were made to the original notes and published reconstructions. Many of these updates were additions of previously missed neuromuscular junctions between ventral cord motor neurons and body wall muscles.

We found that a large section on the dorsal side of the worm, from just anterior to the vulva to the pre-anal ganglion, was never electron micrographed at high power magnification. This dearth of imagery was why so many neurons were missing dorsal side reconstructions. Using original thin sections for the *N2U* worm prepared by White *et al.*, we produced new high power electron micrographs of this dorsal region. Due to the condition of the sections, only one of every 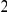
–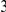 sections was imaged. These new electron micrographs extended nearly 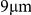 on the dorsal side. New dorsal side data for 3 neurons (DA5, DB4, DD3) were obtained from these electron micrographs. Resource constraints prevented us from covering the entire dorsal gap.

From our compilation of wiring data, including new reconstructions of ventral cord motor neurons, we applied self-consistency criteria to isolate neurons with mismatched reciprocal records. The discrepancies were reconciled by checking against electron micrographs and the laboratory notebooks of White *et al.* Connections in the posterior region of the animal were also cross-referenced with reconstructions published in [10]. Reconciliation involved 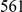 synapses for 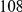 neurons (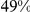 chemical “sends,” 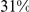 chemical “receives,” and 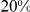 electrical junctions).

### Giant Component for Random Networks

For a random network with 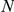 neurons and probability 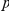 of a connection being present, if the constant 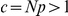, then the size of the giant component is asymptotically normal with mean 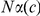 and variance 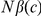 [80, p. 120]. These quantities are given by
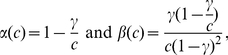
(14)where
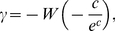
(15)and 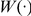 is the Lambert 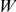-function. If we take 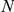 to be 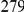 and 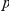 to be 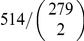, then 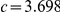. Using the asymptotic approximation, the size of the giant component is distributed approximately normally with mean 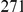 and variance 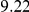. Thus the probability of having a giant component of size 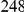, which is over 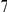 standard deviations from the mean, is about 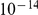. If a precise evaluation of this infinitesimal value is desired, large deviations techniques, rather than the asymptotic approximation may be more valid [81].

To apply this method to the weakly connected component of a directed network, we are interested in the undirected network formed by adding a connection between two neurons if there is a connection in either direction. For a random directed network with probability 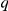 of presence of a directed connection, the probability of a connection existing in either direction is 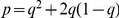. Taking 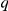 to be 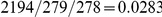, 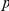 is 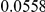. Then for an undirected random network with 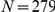 and the specified 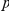, 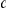 is 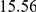. Then the size of the giant component is distributed approximately normally with mean 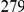 and variance 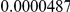. Thus the probability of the giant weakly connected component containing all the neurons in such a random network is overwhelming. Again, large deviations techniques should be used to get a precise evaluation of the probability of being on the order of 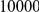 standard deviations away from the mean.

### Giant Component for Random Networks with Given Degree Distribution

Consider the ensemble of random networks with a given degree distribution [82]. For the gap junction network, the generating function corresponding to the measured degree distribution is
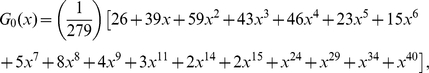
with derivative
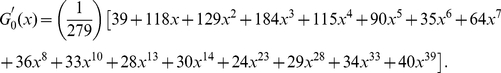
Therefore 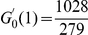. The generating function 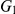 is then
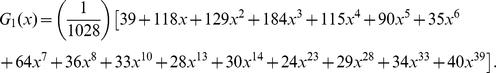
As shown in [82], the expected fraction of the network taken up by the giant component, 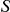, is 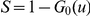, where 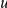 is the smallest non-negative solution to 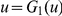. In our case, we find 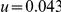, and so 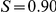. That is to say, one would expect the giant component to consist of 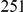 neurons.

Using the computed 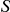 and 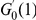, we can find the average component size excluding the giant component, which turns out to be 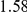.

For the symmetrized chemical network, the generating function corresponding to the measured degree distribution is
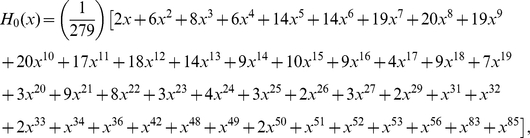
with derivative
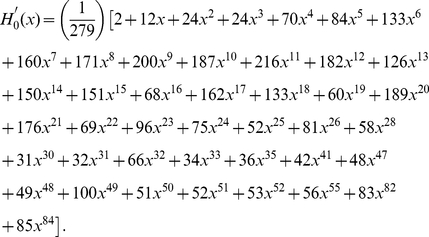
Therefore 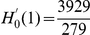. The generating function 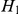 is then
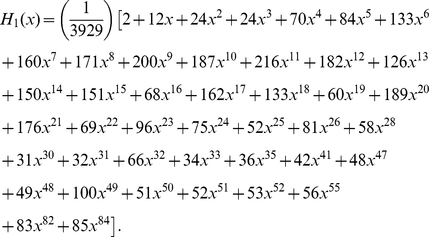



The expected fraction of the network taken up by the giant component, 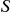, is 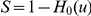, where 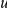 is the smallest non-negative solution to 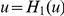. Here 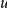 is found to be 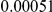, and so 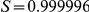. That is to say, one would expect the giant component to consist of 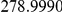 neurons.

### Path Length for Random Networks with Given Degree Distribution

Continuing from the previous subsection, we find the derivative of the generating function 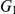 for the gap junction network to be
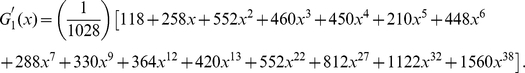
Thus 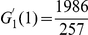. Letting 
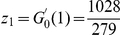
 and 
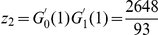
, it is shown in [82, (53)], that the expected path length is
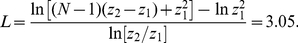
(16)


### Fitting Tails of Distributions

To find functional forms of the tails of various distributions, we follow the procedure outlined in [42]. For the candidate functional forms—say, the power law 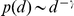 and the exponential decay 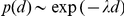
—we perform the following steps. First, we find the optimal parameter of the fit by maximizing the log-likelihood and the optimal starting point of the fit by minimizing the Kolmogorov-Smirnov statistic. Second, we evaluate the goodness of fit by calculating the 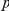-value that the observed data was generated by the optimized distribution using 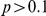 as a criterion for plausibility. Finally, if several distributions pass the 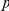-value test we compare their log-likelihoods to find the most probable one.

### Circuits in Eigenmodes

Let us bound the probability of finding an eigenmode that comprises a random set of neurons. Let 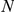 be the number of neurons in the network being analyzed. Let 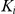 be the number of neurons that appear strongly in the 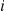
th eigenmode and let 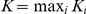. Furthermore let 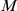 be the number of neurons in the random set, which one might endeavor to investigate as a putative functional circuit derived from an eigenmode.

Now go through each eigenmode and add to a list all possible unordered 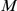-tuples of strong neurons. Even if all of these are unique, the size of the list is upper-bounded by 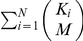 which itself is upper-bounded by 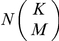.

Additionally, we can compute the number of all unordered 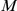-tuples of neurons. This number is 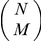.

Thus, if a random set of neurons was selected from all possible sets of neurons, the probability 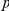 that there would be an eigenmode containing all of them is upper-bounded as
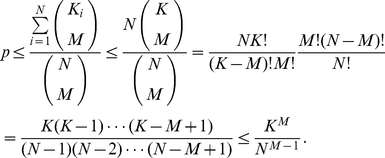



Suppose we are interested in putative functional circuits of size 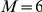 in the giant component of the gap junction network, which has 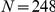 and from Figure 2 in Text S4 take 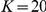. Then even the loosest upper-bound yields
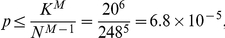
and so finding a random set of neurons in an eigenmode is unlikely.

Suppose we know 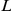 functional circuits of size 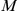 through molecular biology and want to know the probability of at least one of them appearing in the eigenmodes by chance. By the union bound (Boole's inequality), this probability is less than 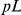. If we take 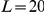 and 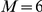, the probability of a known functional circuit appearing in the eigenmodes by chance is less than 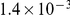 for the giant component of the gap junction network.

### Gap Junction–Chemical Synapse Likelihoods

The likelihood ratios shown in Figure 9 are the following quantities, empirically estimated from either all neuron pairs or pairs with a GABAergic presynaptic neuron. The first is
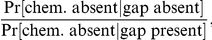
The second is
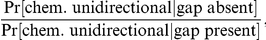
and the third is
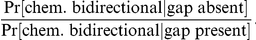



### Degree Correlation Coefficients

Table 1 shows the Pearson correlation coefficients between neuron degree sequences. The average Pearson correlation coefficients of randomly permuted degree sequences from 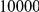 trials are also shown for comparison. The standard deviation is also shown since the distributions of the three randomized correlation coefficients were all nearly symmetric about zero.

### 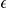-Pseudospectrum Computation

We used the MATLAB package EigTool [83] to compute pseudospectra.

### MATLAB Code and Data

Note that MATLAB code for computing several network properties is available at http://mit.edu/lrv/www/elegans/. This collection of software may be used not only to reproduce most of the figures in this paper, but also for future connectomics analyses.

The collected data is available from the WormAtlas [22] as well as from the same website as the MATLAB code.

## Supporting Information

Text S1 Algorithm for directed network drawing.
(0.09 MB PDF)

Text S2 Algebraic form of survival functions.
(0.09 MB PDF)

Text S3 Eigendecomposition.
(0.16 MB PDF)

Text S4 Supporting figures and tables.
(0.08 MB PDF)
