# Supplementary material for: Structural Properties of the Caenorhabditis elegans Neuronal Network
Source: PLoS Comput Biol. 2011 Feb 3;7(2):e1001066. doi: 10.1371/journal.pcbi.1001066 (PMC3033362; doi:10.1371/journal.pcbi.1001066)
Supplement: Text S1 — Algorithm for directed network drawing. (0.09 MB PDF) [file pcbi.1001066.s001.pdf]

# Text S1: Algorithm for Directed Network Drawing



To visualize a directed neuronal network we modify an approach suggested in [1, 2]. In this approach, the vertical and the horizontal coordinates are chosen independently. The arrangement of neurons along the vertical axis conveys information about the directionality of the signal flow in the network and the arrangement of neurons along the horizontal axis or axes conveys information about the strength of connectivity regardless of directionality.

To find the vertical coordinate, $z$, we try to arrange the neurons so that for every synaptically connected pair of neurons, the difference in $z$ between a presynaptic neuron $i$ and a postsynaptic neuron $j$ is as close to one as possible. Specifically, we minimize the following energy function:

$$E = \frac{1}{2} \sum_{i,j=1}^{n} W_{ij} \left( z_i - z_j - \mathrm{sgn}(A_{ij} - A_{ji}) \right)^2 \tag{1}$$

of the connectivity matrix $A_{ij}$, which is the sum of the gap junction and chemical connectivity matrices, and the symmetrized connectivity matrix $W_{ij}$, which satisfies $W_{ij} = (A_{ij} + A_{ji})/2$. By setting the derivative of this expression to zero, we find:

$$Lz = b, \tag{2}$$

where $b_i = \sum_{j=1}^{n} W_{ij} \mathrm{sgn}(A_{ij} - A_{ji})$ and the Laplacian $L = D - W$ is defined in terms of a diagonal matrix $D$ that contains the number of synaptic terminals on corresponding neurons,

$$D_{ij} = \delta_{ij} \sum_{k=1}^{n} W_{ik}. \tag{3}$$

A unique solution to this equation can be found by using the pseudoinverse.

To find the horizontal coordinates, we use the Laplacian, $L$, normalized by the number-of-terminals matrix $D$,

$$Q = D^{-1/2} L D^{-1/2}. \tag{4}$$

The eigenmodes corresponding to the second and third lowest eigenvalues of $Q$ are denoted $v_2$ and $v_3$. Then, the horizontal coordinates are

$$x = D^{-1/2} v_2 \quad \text{and} \quad y = D^{-1/2} v_3. \tag{5}$$

This method produces an aesthetically appealing drawing because each neuron is placed in the weighted centroid of its neighbors. Thus strongly coupled neurons tend to be colocated.

## References

1. Carmel L, Harel D, Koren Y (2004) Combining hierarchy and energy for drawing directed graphs. IEEE Trans Vis Comput Graphics 10:46–57. doi:10.1109/TVCG.2004.1260757.

2. Koren Y (2005) Drawing graphs by eigenvectors: Theory and practice. Comput Math Appl 49:1867–1888. doi:10.1016/j.camwa.2004.08.015.
