# Supplementary material for: Structural Properties of the Caenorhabditis elegans Neuronal Network
Source: PLoS Comput Biol. 2011 Feb 3;7(2):e1001066. doi: 10.1371/journal.pcbi.1001066 (PMC3033362; doi:10.1371/journal.pcbi.1001066)
Supplement: Text S2 — Algebraic form of survival functions. (0.09 MB PDF) [file pcbi.1001066.s002.pdf]

Text S2: Algebraic Form of Survival Functions

Here we consider several commonly encountered distributions and their survival functions. If a distribution were to follow a power law, $p(d) \sim d^{-\alpha}$, then the survival function (under a continuous approximation) also follows a power law:

$$P(d) = \sum_{k=d}^{\infty} k^{-\alpha} \sim d^{-(\alpha-1)}. \tag{1}$$

Similarly, if a distribution follows an exponential decay, $p(d) \sim e^{-d/\kappa}$, then the survival function also has an exponential decay, with the same exponent:

$$P(d) = \sum_{k=d}^{\infty} e^{-k/\kappa} \sim e^{-d/\kappa}. \tag{2}$$

If a distribution were to follow the (continuous) stretched exponential distribution, $p(d) \sim (d/\beta)^{\gamma-1} e^{-(d/\beta)^{\gamma}}$, then the survival function would have a decay given by a stretched exponential function with the same stretch factor γ:

$$P(d) = \sum_{k=d}^{\infty} (k/\beta)^{\gamma-1} e^{-(k/\beta)^{\gamma}} \sim e^{-(d/\beta)^{\gamma}} \tag{3}$$

[1].

References

1. Clauset A, Shalizi CR, Newman MEJ (2009) Power-law distributions in empirical data. SIAM Rev 51:661–703. doi:10.1137/070710111.
