# Supplementary material for: Structural Properties of the Caenorhabditis elegans Neuronal Network
Source: PLoS Comput Biol. 2011 Feb 3;7(2):e1001066. doi: 10.1371/journal.pcbi.1001066 (PMC3033362; doi:10.1371/journal.pcbi.1001066)
Supplement: Text S3 — Eigendecomposition. (0.16 MB PDF) [file pcbi.1001066.s003.pdf]

Text S3: Eigendecomposition

Physical systems are often represented by linear, constant-coefficient differential equations. Differential equations provide an implicit specification of the system, giving the relationship between input and output, rather than an explicit expression for the system output as a function of the input. After specifying initial conditions, differential equations can be solved to find explicit expressions for the output.

Dynamical systems that can store energy in only one form and location are called *first-order*, since the equation describing time evolution can be written only in terms of a single variable and its first derivative. Storing energy is a form of short-term memory. For a single state variable V_i, a canonical first-order, linear, constant-coefficient differential equation is

$$\tau \frac{dV_i(t)}{dt} + L_{ii}V_i(t) = M(t),$$

where τ and L_{ii} are fixed constants and $M(t)$ is some signal.

The natural (unforced) response of a system corresponds to $M(t) = 0$ and is completely determined by the system's *eigenvalue*. In particular, solving

$$\tau \frac{dV_i(t)}{dt} + L_{ii}V_i(t) = 0$$

with initial condition $V_i(t = 0) = V_0$, yields

$$V_i(t) = V_0 e^{(-L_{ii}/\tau)t},$$

where $-L_{ii}/\tau$ is the eigenvalue.

The forced response occurs when some exogenous perturbation is applied to the system. For example if a scaled step function $M_0u(t)$ is applied, then the differential equation

$$\tau \frac{dV_i(t)}{dt} + L_{ii}V_i(t) = M_0u(t)$$

with initial condition $V_i(t = 0) = V_0$ has solution

$$V_i(t) = \left\{ \frac{M_0}{L_{ii}} + \left[V_0 - \frac{M_0}{L_{ii}} \right] e^{(-L_{ii}/\tau)t} \right\},\ t > 0.$$

The response of a first-order system to a unit impulse is identical to its natural response; the impulse generates the initial condition in such a short time that it has no other effect on the system. That is, the system is jarred to the initial position by the impulse.

Generally when a forcing function is applied to a linear constant-coefficient dynamic system, the response will consist of the superposition of the forced response (a modification of the input signal) and the natural response governed by the system's eigenproperties.

Thus far, we have considered a single state variable $V_i(t)$, but in neuronal networks we actually have a vector of states, $V(t) = \begin{bmatrix} V_1(t) & V_2(t) & \cdots & V_N(t) \end{bmatrix}^T$, governed by a system of linear constant-coefficient differential equations. A canonical form is

$$\begin{aligned}
\tau \frac{dV_1(t)}{dt} + L_{11}V_1(t) + L_{12}V_2(t) + \cdots + L_{1N}V_N(t) &= M_1(t) \\
\tau \frac{dV_2(t)}{dt} + L_{21}V_1(t) + L_{22}V_2(t) + \cdots + L_{2N}V_N(t) &= M_2(t) \\
\vdots \qquad\qquad &= \ \vdots \\
\tau \frac{dV_N(t)}{dt} + L_{N1}V_1(t) + L_{N2}V_2(t) + \cdots + L_{NN}V_N(t) &= M_N(t)
\end{aligned}$$

which can be written in matrix-vector form as

$$\tau\frac{dV(t)}{dt} + LV(t) = M(t).$$

The natural response of such a system with initial condition $V(t=0) = V_0$ is the vector

$$V(t) = V_0 e^{(-L/\tau)t}.$$

Although this is in principle the solution to the system of differential equations, it is difficult to examine. Study of system behavior is complicated by the fact that each of the equations is coupled to the others through the off-diagonal elements of L. It would be desirable to find a new coordinate system in which all equations are decoupled (such that the coefficient matrix is diagonal).

A vector v is called an eigenmode of a matrix L if it satisfies

$$Lv = \lambda v$$

for some number λ, which is called the eigenvalue. Decomposing the coefficient matrix into its eigendecomposition,[1]

$$L = \begin{bmatrix} v_1 & v_2 & \cdots & v_N \end{bmatrix} \begin{bmatrix} \lambda_1 & 0 & \cdots & 0 \\ 0 & \lambda_2 & \cdots & 0 \\ \vdots & \vdots & \ddots & \vdots \\ 0 & 0 & \cdots & \lambda_N \end{bmatrix} \begin{bmatrix} v_1 & v_2 & \cdots & v_N \end{bmatrix}^{-1}$$

allows us to write the natural response as

$$V(t) = \sum_{i=1}^{N} v_i \alpha_i e^{(-\lambda_i/\tau)t},$$

where α_i is the projection of the initial condition vector V_0 onto v_i.

The essential idea of the eigenmode decomposition is that the natural response of the system can be viewed as the superposition of a number of distinct types of dynamics—the eigenmodes—each one associated with a particular natural frequency of the system. The natural frequencies, $-\lambda_i/\tau$, of the system are determined by the eigenvalues λ_i of L. Each mode involves excitation of one and only one natural frequency of the system.

If an eigenmode is real, then the dynamics associated with the solution can be described by a straight line in state space. The system moves in the direction of the eigenmode. For example, moving in the direction of the eigenmode $\begin{bmatrix} +1 & -1 & 0 & 0 & 0 & \cdots & 0 \end{bmatrix}^T$ would equalize the values of V_1 and V_2 but not affect $V_3, \ldots, V_N$. A more complicated eigenmode would involve all state variables that are non-zero.

Beyond their simple geometric interpretation in state space, the eigenmodes also have a simple representation as time functions, since each one involves a single exponential rather than a mixture of several exponentials with different exponents. The exponent $-\lambda_i/\tau$ determines how quickly the system response in the direction of eigenmode v_i decays. For fixed τ, the larger the eigenvalue λ_i, the more quickly the eigenmode decays.

The forced response of a network proceeds in the same way as the forced response of a scalar system. Further details on linear system analysis with eigenmodes can be found, e.g. in the textbooks [3, 4].

---

[1] Note that not all matrices have an eigendecomposition. Instead, the Jordan decomposition should be used for these non-diagonalizable matrices [1]. The three matrices we consider, L, A^T, and Φ are diagonalizable and so the eigendecomposition is identical to the Jordan decomposition.

Another decomposition that has been proposed for use in systems neuroscience is the Schur decomposition [2]. Since the gap junction network is undirected, the Schur decomposition is also identical to the eigendecomposition. For the chemical and combined networks, the Schur modes may provide additional insights, but we do not consider them in this work.

References

1. Nishikawa T, Motter AE (2006) Synchronization is optimal in nondiagonalizable networks. Phys Rev E 73:065106. doi:10.1103/PhysRevE.73.065106.

2. Goldman MS (2009) Memory without feedback in a neural network. Neuron 61:621–634. doi: 10.1016/j.neuron.2008.12.012.

3. Edwards CH, Penney DE (2000) Differential Equations and Boundary Value Problems: Computing and Modeling. Upper Saddle River, NJ: Prentice Hall.

4. Stern TE (1965) Theory of Nonlinear Networks and Systems: An Introduction. Reading, MA: Addison-Wesley.
