# Supplementary material for: Structural Properties of the Caenorhabditis elegans Neuronal Network
Source: PLoS Comput Biol. 2011 Feb 3;7(2):e1001066. doi: 10.1371/journal.pcbi.1001066 (PMC3033362; doi:10.1371/journal.pcbi.1001066)
Supplement: Text S4 — Supporting figures and tables. (0.08 MB PDF) [file pcbi.1001066.s004.pdf]

Text S4: Supporting Figures and Tables

Table 1. Connected components of the gap junction network. Note the single giant component and the large number of disconnected/isolated neurons.

Giant Component (248 neurons)

ADAL/R	ALNL	AVG	DD01-05	PDA	PVR	RIVL/R	SABVL/R	URYVL/R
ADEL/R	AQR	AVHL/R	DVA	PDB	PVT	RMDDL/R	SDQL/R	VA01-12
ADFL/R	AS01-11	AVJL/R	DVB	PDEL/R	PVWL/R	RMDL/R	SIADL/R	VB01-11
ADLL/R	ASGL/R	AVKL/R	DVC	PHAL/R	RIBL/R	RMDVL/R	SIAVL/R	VC01-05
AFDL/R	ASHL/R	AVL	FLPL/R	PHBL/R	RICL/R	RMED	SIBDL/R	VD01-10,13
AIAL/R	ASIL/R	AVM	IL1DL/R	PHVL/R	RID	RMEL/R	SIBVL/R	
AIBL/R	ASKL/R	AWAL/R	IL1L/R	PLML/R	RIFL/R	RMEV	SMBDL/R	
AIML	AUAL/R	AWBL/R	IL1VL/R	PQR	RIGL/R	RMFL	SMBVL/R	
AINL/R	AVAL/R	BAGL/R	IL2L/R	PVCL/R	RIH	RMGL/R	SMDDL/R	
AIYL/R	AVBL/R	CEPDL/R	LUAL/R	PVM	RIML/R	RMHL/R	SMDVL/R	
AIZL/R	AVDL/R	CEPVL/R	OLLL/R	PVNL	RIPL/R	SAADL/R	URBL/R	
ALA	AVEL/R	DA01-09	OLQDL/R	PVPL/R	RIR	SAAVL/R	URXL/R	
ALML/R	AVFL/R	DB01-07	OLQVL/R	PVQL/R	RIS	SABD	URYDL/R	

First Small Component (2 neurons)

ASJL/R

Second Small Component (3 neurons)

HSNL/R	PVNR

Neurons with no gap junctions (26 neurons)

AIMR	ASEL/R	BDUL/R	IL2DL/R	PLNL/R	RIAL/R	URADL/R	VD11-12
ALNR	AWCL/R	DD06	IL2VL/R	PVDL/R	RMFR	URAVL/R	

Table 2. (A) Number of gap junction contacts between different neuron categories. (B) Percent of gap junctions on neurons of the row category that connect to neurons of the column category.

**A**	Sensory	Inter-	Motor
Sensory	108	119	26
Inter-	119	368	342
Motor	26	342	324

**B**	Sensory	Inter-	Motor
Sensory	42.7%	47.0%	10.3%
Inter-	14.4%	44.4%	41.3%
Motor	3.8%	49.4%	46.8%

Table 3. (A) Number of chemical synapse contacts from row category to column category. (B) Percent of synapses in row category that synapse to column category.

**A**	Sensory	Inter-	Motor
Sensory	474	1434	353
Inter-	208	1359	929
Motor	30	275	1332

**B**	Sensory	Inter-	Motor
Sensory	21.0%	63.4%	15.6%
Inter-	8.3%	54.5%	37.2%
Motor	1.8%	16.8%	81.4%

Table 4. Strongly connected components of the chemical network. Note the single giant component and the large number of isolated neurons.

Giant Component (237 neurons)

ADAL/R	ALNL/R	AVFL/R	CEPVL/R	LUAL/R	PVM	RIH	RMHL/R	URYDL/R
ADEL/R	AQR	AVG	DA01-06,09	OLLL/R	PVNL/R	RIML/R	SAADL/R	URYVL/R
ADFL/R	AS01-06,09,11	AVHL/R	DB01-04,07	OLQDL/R	PVPL/R	RIPL/R	SAAVL/R	VA01-06,08-09,11-12
ADLL/R	ASEL/R	AVJL/R	DD01-02,05	OLQVL/R	PVQL/R	RIR	SABD	VB01-06,08-11
AFDL/R	ASGL/R	AVKL/R	DVA	PDA/B	PVR	RIS	SDQL	VC01-05
AIAL/R	ASHL/R	AVL	DVC	PDEL/R	PVT	RIVL/R	SMBDL/R	VD01-03,05-06,08,10-13
AIBL/R	ASJL/R	AVM	FLPL/R	PHAL/R	PVWL/R	RMDDR	SMBVL/R	
AIML/R	ASKL/R	AWAL/R	HSNL/R	PHBL/R	RIAL/R	RMDL/R	SMDDL/R	
AINR	AUAL/R	AWBL/R	IL1DL/R	PLMR	RIBL/R	RMDVL	SMDVL/R	
AIYL/R	AVAL/R	AWCL/R	IL1L/R	PLNL	RICL/R	RMED	URADL/R	
AIZL/R	AVBL/R	BAGL/R	IL1VL/R	PQR	RID	RMEV	URAVL/R	
ALA	AVDL/R	BDUL/R	IL2L/R	PVCL/R	RIFL/R	RMFL/R	URBL/R	
ALML/R	AVEL/R	CEPDL/R	IL2VL/R	PVDL	RIGL/R	RMGL/R	URXL/R	

Small Component (2 neurons)

RMDVR	RMDDL

Isolated neurons in chemical network (40 neurons)

AINL	DA07-08	DVB	PLML	RMEL/R	SDQR	SIAVL/R	SIBVL/R	VB07
ASIL/R	DB05-06	IL2DL/R	PLNR	SABVL/R	SIADL/R	SIBDL/R	VA07,10	VD04,07,09
AS07,08,10	DD03-04,06	PHCL/R	PVDR					

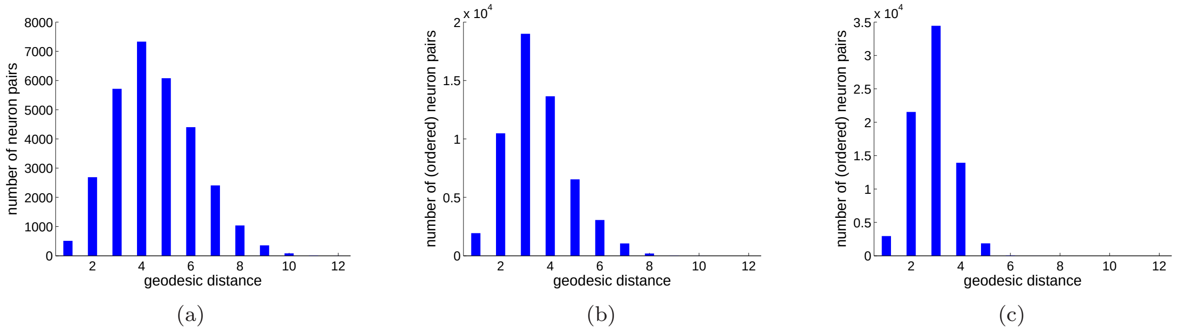

Figure 1. Geodesic distance distributions. (a). Giant component of gap junction network. (b). Giant component of chemical network. (c). Giant component of combined network.

Table 5. Some structural properties of the *C. elegans* gap junction network, randomly edited networks (E_{gap}), and the AY network [1].

	C. elegans	AY's *C. elegans* [1]	E_{gap}
d_{edit}	—	454	177 ± 18.5
giant component size	248	253	261 ± 3.41
giant component pathlength	4.52	4.71	4.09 ± 0.078
giant component clust. coef.	0.21	0.23	0.14 ± 0.011

References

1. Achacoso TB, Yamamoto WS (1992) AY's Neuroanatomy of *C. elegans* for Computation. CRC Press.

Table 6. Some structural properties of the *C. elegans* chemical network, randomly edited networks (E_{chem}), and the AY network [1].

	C. elegans	AY's *C. elegans* [1]	E_{chem}
d_{edit}	—	3546	638 ± 33.2
weak giant component size	279	279	279 ± 0.07
strong giant component size	237	239	267 ± 3.19
strong giant component pathlength	3.48	3.99	3.12 ± 0.028
strong giant component clust. coef.	0.22	0.20	0.16 ± 0.006

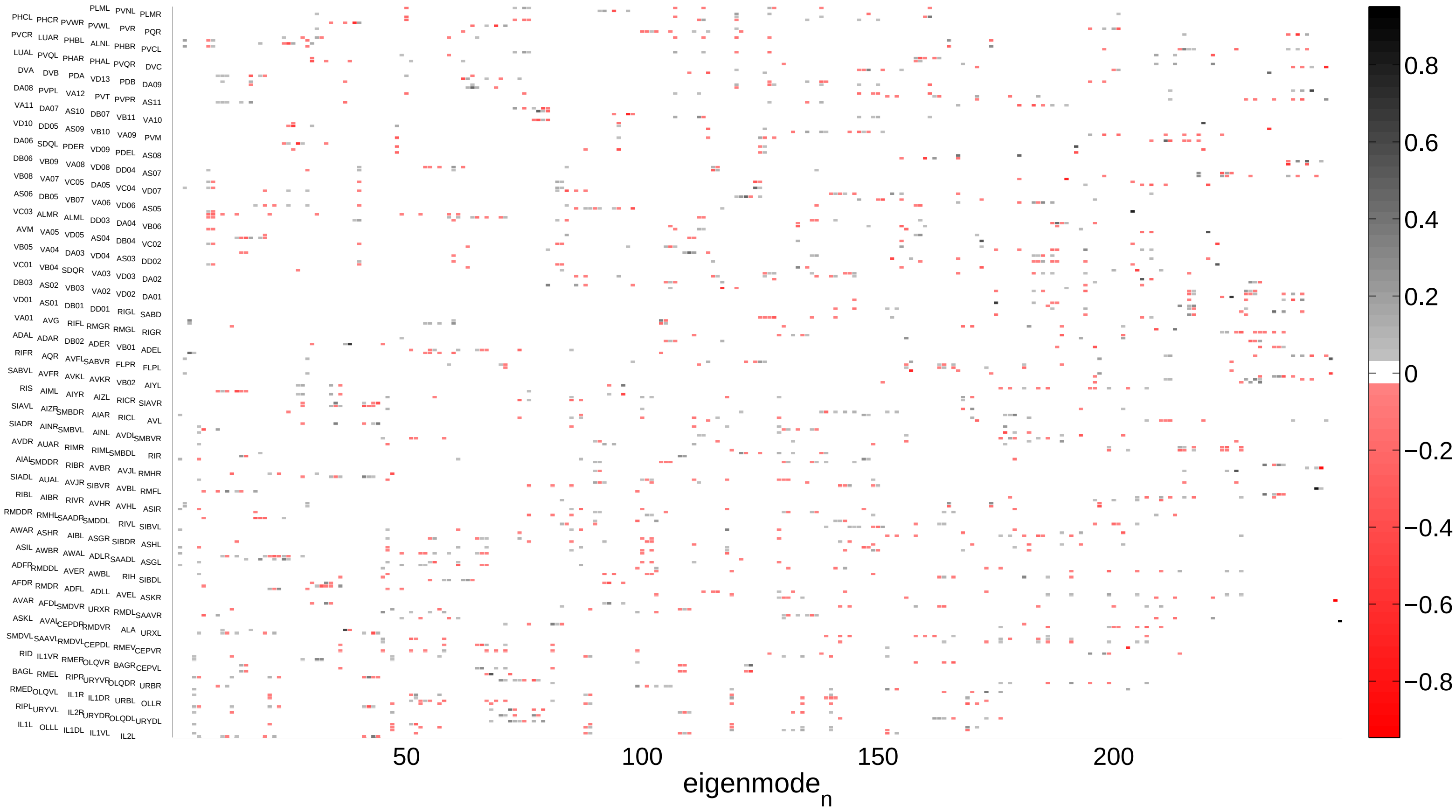

Figure 2. Eigenmodes of Laplacian for giant component of gap junction network.

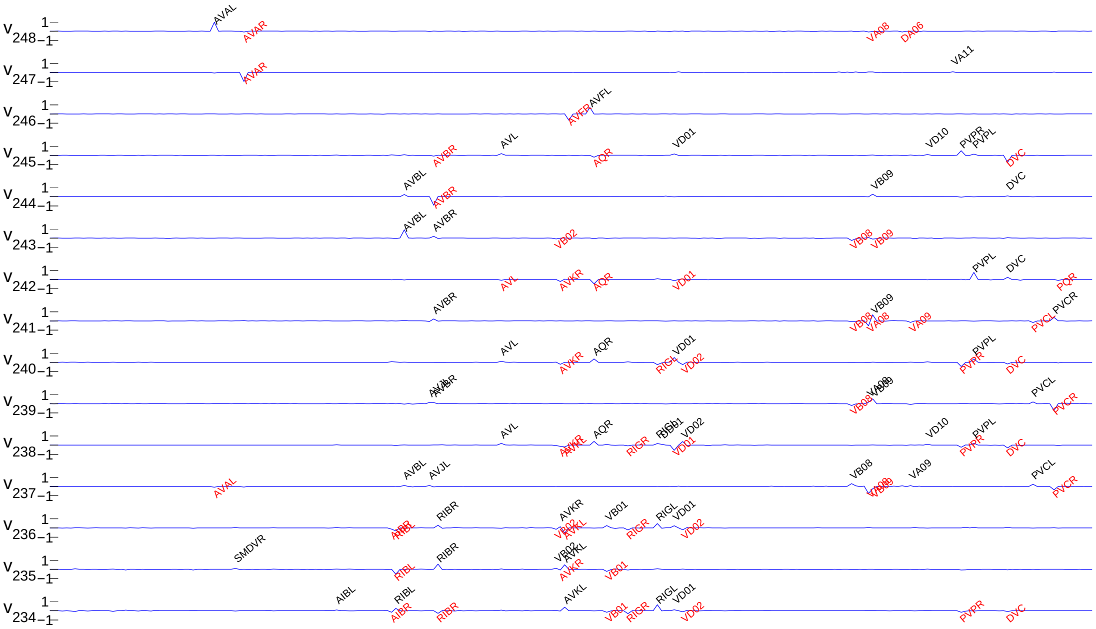

Figure 3. Fastest eigenmodes of Laplacian for giant component of gap junction network. Eigenmodes corresponding to $\lambda_{248}, \lambda_{247}, \ldots, \lambda_{234}$ are shown. The eigenmodes are labeled with neurons that take value above a fixed absolute value threshold. Neurons with negative values are in red, whereas neurons with positive values are in black.

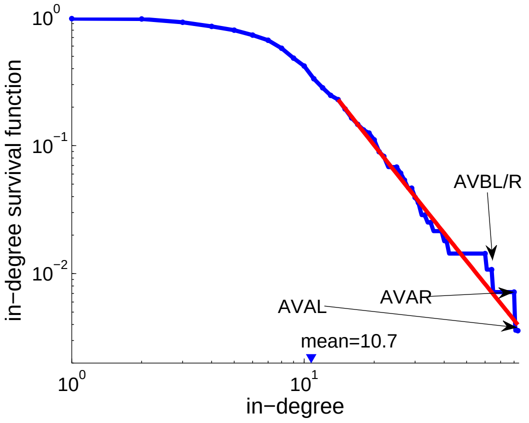

(a)

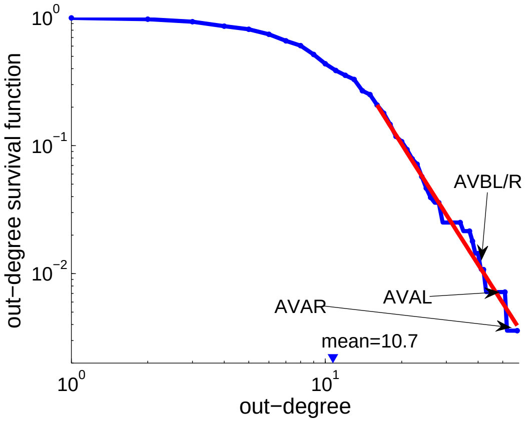

(b)

Figure 4. Survival functions of the in-degree (a) and out-degree (b) distributions in the combined network. The tails of the distributions can be fit with power laws.
